# The effect of shortening the quarantine period and lifting the indoor mask mandate on the spread of COVID-19: a mathematical modeling approach

**DOI:** 10.3389/fpubh.2023.1166528

**Published:** 2023-07-21

**Authors:** Jung Eun Kim, Heejin Choi, Minji Lee, Chang Hyeong Lee

**Affiliations:** ^1^Department of Mathematics and Computer Science, Korea Science Academy of KAIST, Busan, Republic of Korea; ^2^Department of Mathematical Sciences, Ulsan National Institute of Science and Technology, Ulsan, Republic of Korea

**Keywords:** COVID-19, quarantine, mask wearing, mathematical modeling, cost-effectiveness

## Abstract

In this paper, we present a mathematical model to assess the impact of reducing the quarantine period and lifting the indoor mask mandate on the spread of Coronavirus Disease 2019 (COVID-19) in Korea. The model incorporates important epidemiological parameters, such as transmission rates and mortality rates, to simulate the transmission of the virus under different scenarios. Our findings reveal that the impact of mask wearing fades in the long term, which highlights the crucial role of quarantine in controlling the spread of the disease. In addition, balancing the confirmed cases and costs, the lifting of mandatory indoor mask wearing is cost-effective; however, maintaining the quarantine period remains essential. A relationship between the disease transmission rate and vaccine efficiency was also apparent, with higher transmission rates leading to a greater impact of the vaccine efficiency. Moreover, our findings indicate that a higher disease transmission rate exacerbates the consequences of early quarantine release.

## 1. Introduction

The social distancing policy in Korea, which was implemented on March 22, 2020, lasted until April 17, 2022, with various changes in the guidance (1). On April 25, 2022, Coronavirus Disease 2019 (COVID-19) was lowered from Class 1, which requires a high level of isolation, such as negative-pressure isolation, to Class 2, which maintains only 7 days isolation for confirmed cases (2). In addition, since the outdoor mask mandate was lifted on September 26, 2022, the primary nonpharmaceutical interventions (NPIs) maintained in Korea were the indoor mask mandate and 7 days quarantine. However, as COVID-19 has continued for more than 2 years, there are calls to shorten the quarantine duration and ease mandatory indoor mask wearing due to people’s fatigue and for economic reasons. In this study, we analyzed the number of confirmed cases, number of severe cases, and the economic impact according to the change in the current NPIs.

As COVID-19 spread across the world, the COVID-19 dynamics, such as the number of confirmed cases and death cases, were predicted using mathematical models. After the development of the vaccine, a mathematical model that considered the vaccine efficacy and rollout was used to simulate the impact of vaccination (3, 4). In addition, to prevent breakthrough infections and large-scale spread due to variants, many countries recommended boosted vaccination (third vaccination), and the Korean government also recommended a fourth vaccination and bivalent vaccination for high-risk groups. Bosetti et al. (5), Ngonghala et al. (6), and Gavish et al. (7) examined whether the boosted vaccination helped to eliminate COVID-19 using a mathematical model that included a population group that had received this vaccination.

Because the infection probability gradually decreases after the peak (8–10), many countries have implemented various quarantine policies to prevent the spread of COVID-19. Zhang et al. (11) demonstrated that the self-isolation of susceptible people is effective at reducing the effective reproduction number, and Yu et al. (12) showed that if early testing is impossible, then the isolation of symptomatic people is necessary. In Ashcroft et al. (13), the effect on the transmission due to the reduction in the duration of quarantine for people who returned from abroad was investigated. In Ma et al. (14) and Lindsley et al. (15), the efficacy of facial masks for blocking the SARS-CoV-2 virus was demonstrated in experiments, and the effect of facial masks on COVID-19 transmission was studied in Ngonghala et al. (6), Motallebi et al. (16), Shen et al. (17), and Reiner et al. (18). Further, Baek et al. (19) and Kim et al. (20) studied the transmission according to indoor mask wearing in a specific group in Korea. In addition, there were several studies to analyze the economic impact of COVID-19. Li et al. (21) showed that it is cost-effective to inoculate the booster vaccine to seniors aged 65 years or older, even considering the cost of booster vaccination. Kim et al. (22) analyzed cost-effectiveness according to the social distancing level and vaccine supply speed. Along with vaccine developments, COVID-19 treatment was also developed, so Jo et al. (23) studied the cost-effectiveness of COVID-19 treatments, namely Remdesivir and Dexamethasone.

In this paper, we develop a mathematical model to examine the impact of easing COVID-19 control measures, such as the quarantine and mask-wearing requirements. The model considers the effects of vaccination and reinfection, and its parameters were estimated accordingly. We used this model to explore the changes in confirmed cases, hospitalizations, and deaths over time according to various quarantine durations and mask-wearing rates. Furthermore, we investigated the impact of quarantine and mandatory mask wearing from a cost-effectiveness perspective.

## 2. Methods

### 2.1. Epidemiological data

Since January 19, 2020, when the first case of COVID-19 was confirmed, the total number of confirmed cases in Korea has gradually increased. The control strategy in Korea received good press before the inflow of the Omicron variant. However, after the inflow of Omicron, which has higher transmissibility than previous variants or the wild type due to its higher stability in all open-complex forms, the number of confirmed COVID-19 cases in Korea exploded (24).

**Figure 1:**
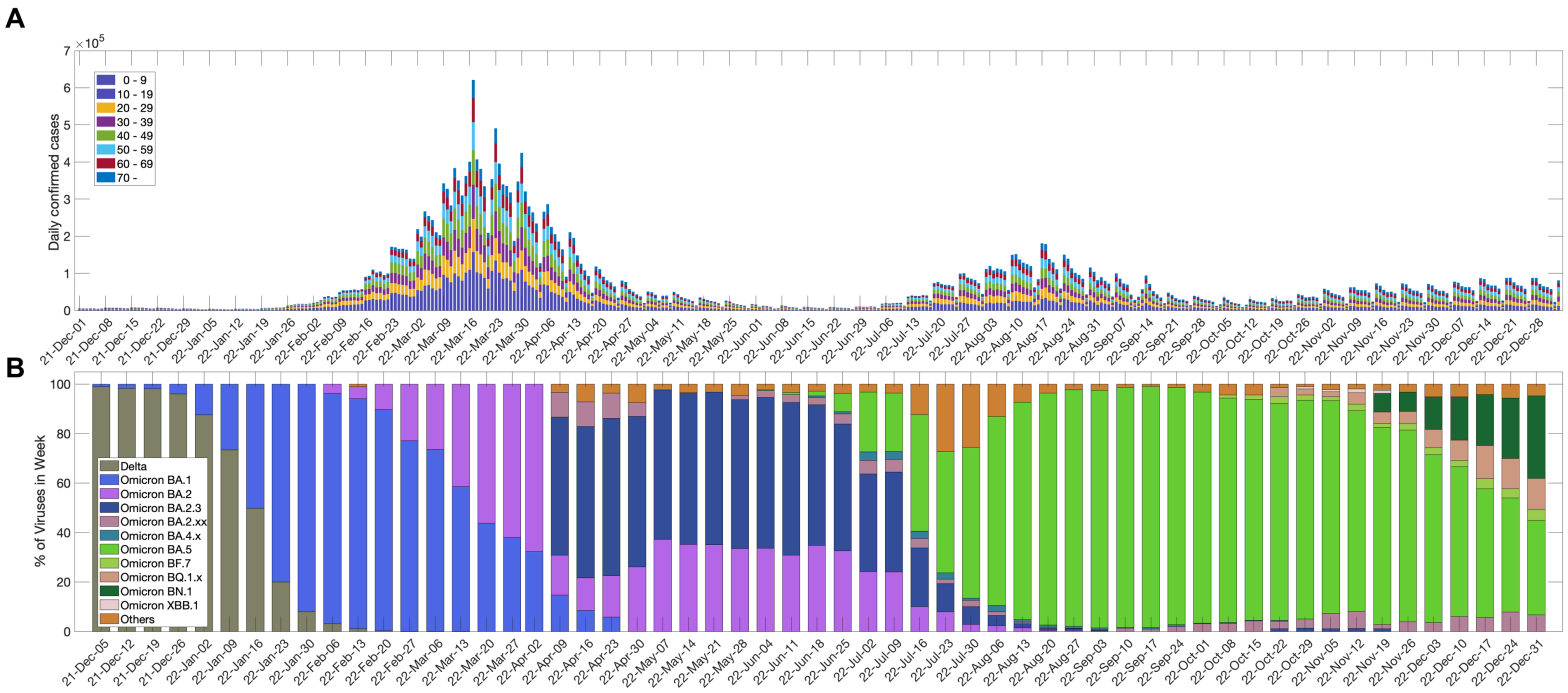
**(A)** Daily confirmed cases in each age group; **(B)** percentage of SARS-CoV-2 variants of concern.

Figure 1A shows the daily confirmed cases in each age group after the inflow of the Omicron variant. On March 17, 2022, there was a large wave of more than 600,000 confirmed cases per day, and in August 2022, there was another large wave. Since November 2022, the number of confirmed cases due to the variant has gradually increased.

The Korea Disease Control and Prevention Agency (KDCA) announced COVID-19 antibody positive rate survey results using data on September 6, 2022. According to these results, more than 97% of Koreans have COVID-19 antibodies from infections or vaccinations, and the antibody rate from infection was reported to be 57.65%, which is higher than the cumulative infection rate of 38.15% that was measured at the same time, which means there were many unreported infected people in the local society (25). Even though Koreans have a high antibody rate, the epidemic is not abating due to factors such as waning immunity and new variants. Figure 1B shows the weekly detection rate for each variant of the SARS-CoV-2 virus since December 2021. The Omicron variant was dominant in Korea on January 16, 2022, and since then, several Omicron subvariants have appeared.

### 2.2. Mathematical model

In this study, we used an age-structured mathematical model to describe the COVID-19 dynamics in Korea across eight age groups: 0–9 years; 10–19 years; 20–29 years; 30–39 years; 40–49 years; 50–59 years; 60–69 years; 70 years and older.

We considered different factors to develop the mathematical model that fit the Korean COVID-19 situation. First, we divided the vaccinated population into compartments for the second vaccination, third vaccination, and fourth vaccination. Here, the second vaccination, third vaccination, and fourth vaccination refer to the second dose of the first vaccination, the first booster vaccination after the second dose, and the second booster vaccination after the second dose, respectively. We categorized the first-vaccinated population as susceptible, taking into account that the efficacy of the first vaccination is almost negligible on the initial date of our model simulation (26), which is February 1, 2022, almost 1 year after the first vaccine administration in Korea in February 2021. The Korean government has recommended up to a fourth vaccination for the population aged 50 years and older and a third vaccination for the population aged 18–49 years (27). Second, the population that tested positive for COVID-19 and needed to be quarantined was categorized according to the symptoms of its members: mild-symptom cases, hospitalization without intensive care, and hospitalization with intensive care. Some members of the population who were released from intensive care units were moved to the general ward, represented by $F^{H},F^{HV}$ . Moreover, we considered the members of the unreported infected population, who can infect other susceptible populations and who have mild symptoms or are asymptomatic. Third, we considered the reinfection rate and the waning of the vaccination efficacy. Because the COVID-19 pandemic has continued for a long time, the reinfection rate and breakthrough infection rate are increasing. Lastly, to investigate the effect of the quarantine duration, we added the confirmed cases of early quarantine release as $F^{M},F^{MV}$ . Because we assumed that patients who were released early (before 7 days) were still infectious, they affected the infection rate. By taking into account the above information, we can write the equations for the mathematical model as follows;


$$\dot{S_{i}}=-\Lambda_{i}S_{i}-v_{2i}$$

$$\dot{E_{i}}=\Lambda_{i}S_{i}-\alpha E_{i}$$

$$\dot{I_{i}}=\alpha \left(E_{i}+E_{i}^{R}\right)-q\left(\delta_{i}^{M}+\delta_{i}^{H}+\delta_{i}^{I}\right)I_{i}$$

$$\dot{H_{i}^{M}}=q\left(1-\rho \right)\delta_{i}^{M}I_{i}-\gamma^{M}H_{i}^{M}$$

$$\dot{Q_{i}^{M}}=q\rho \delta_{i}^{M}I_{i}-\eta^{Q}Q_{i}^{M}$$

$$\dot{F_{i}^{M}}=\eta^{Q}Q_{i}^{M}-\eta^{F}F_{i}^{M}$$

$$\dot{Q_{i}^{H}}=q\delta_{i}^{H}I_{i}-\gamma_{i}^{H}Q_{i}^{H}$$

$$\dot{Q_{i}^{I}}=q\delta_{i}^{I}I_{i}-\eta^{I}\left(1-\kappa_{i}\right)Q_{i}^{I}-\gamma_{i}^{I}\kappa_{i}Q_{i}^{I}$$

$$\dot{F_{i}^{H}}=\eta^{I}\left(1-\kappa_{i}\right)Q_{i}^{I}-\zeta^{I}F_{i}^{H}$$

$$\dot{V_{i}^{2}}=-\Lambda_{i}\left(1-\tau_{2}\right)V_{i}^{2}+v_{2i}-v_{3i}$$

$$\dot{V_{i}^{3}}=-\Lambda_{i}\left(1-\tau_{3}\right)V_{i}^{3}+v_{3i}-v_{4i}$$

$$\dot{V_{i}^{4}}=-\Lambda_{i}\left(1-\tau_{4}\right)V_{i}^{4}+v_{4i}$$

$$\dot{E_{i}^{V}}=\Lambda_{i}\left(\left(1-\tau_{2}\right)V_{i}^{2}+\left(1-\tau_{3}\right)V_{i}^{3}+\left(1-\tau_{4}\right)V_{i}^{4}\right)-\alpha E_{i}^{V}$$

$$\dot{I_{i}^{V}}=\alpha E_{i}^{V}-q\left(\delta_{i}^{MV}+\delta_{i}^{HV}+\delta_{i}^{IV}\right)I_{i}^{V}$$

$$\dot{H_{i}^{MV}}=q\left(1-\rho \right)\delta_{i}^{MV}I_{i}^{V}-\gamma^{M}H_{i}^{MV}$$

$$\dot{Q_{i}^{MV}}=q\rho \delta_{i}^{MV}I_{i}^{V}-\eta^{Q}Q_{i}^{MV}$$

$$\dot{F_{i}^{MV}}=\eta^{Q}Q_{i}^{MV}-\eta^{F}F_{i}^{MV}$$

$$\dot{Q_{i}^{HV}}=q\delta_{i}^{HV}I_{i}^{V}-\gamma_{i}^{H}Q_{i}^{HV}$$

$$\dot{Q_{i}^{IV}}=q\delta_{i}^{IV}I_{i}^{V}-\eta^{I}\left(1-\kappa_{i}^{V}\right)Q_{i}^{IV}-\gamma_{i}^{I}\kappa_{i}^{V}Q_{i}^{IV}$$

$$\dot{F_{i}^{HV}}=\eta^{I}\left(1-\kappa_{i}^{V}\right)Q_{i}^{IV}-\zeta^{I}F_{i}^{HV}$$

$$\begin{array}{l} \dot{R_{i}}=\gamma^{M}\left(H_{i}+H_{i}^{MV}\right)+\eta^{F}\left(F_{i}^{M}+F_{i}^{VM}\right)\\ \;\;\;\;\;\;\;+\gamma_{i}^{H}\left(Q_{i}^{H}+Q_{i}^{HV}\right)+\zeta^{I}\left(F_{i}^{H}+F_{i}^{HV}\right)-\psi R_{i} \end{array}$$

$$\dot{S_{i}^{R}}=\psi R_{i}-\Lambda_{i}S_{i}^{R}$$

$$\dot{E_{i}^{R}}=\Lambda_{i}S_{i}^{R}-\alpha E_{i}^{R}$$

$$\dot{D_{i}}=\gamma_{i}^{I}\left(\kappa_{i}Q_{i}^{I}+\kappa_{i}^{V}Q_{i}^{IV}\right)$$

where $\Lambda_{i}=\overset{8}{\underset{k=1}{\sum }}\left[\beta_{ik}\left(\left(I_{k}+I_{k}^{V}\right)+\left(H_{k}+H_{k}^{V}\right)+\theta_{F}\left(F_{k}^{M}+F_{k}^{MV}\right)\right)/N_{k}\right]$ for $N_{k}=S_{k}+E_{i}+H_{i}+I_{i}+F_{i}^{M}+V_{i}^{2}+V_{i}^{3}+V_{i}^{4}+E_{i}^{V}+H_{i}^{V}+I_{i}^{V}+F_{i}^{MV}+R_{i}+E_{i}^{R},$ and the dot above a variable denotes the time derivative of the variable i.e., $\dot{X}=\frac{dX}{dt}$ . In the above equations, $S_{i}\left(t\right)$ denotes susceptible including first-dose vaccinated; $E_{i}\left(t\right)$ denotes unvaccinated and exposed; $I_{i}\left(t\right)$ denotes unvaccinated and infectious; $H_{i}\left(t\right)$ denotes hidden infections without antibodies; $Q_{i}^{M}\left(t\right)$ denotes unvaccinated quarantined mild-symptom patient; $F_{i}^{M}\left(t\right)$ denotes unvaccinated nonquarantined mild-symptom patient; $Q_{i}^{H}\left(t\right)$ denotes unvaccinated hospitalization without intensive care; $Q_{i}^{I}\left(t\right)$ denotes unvaccinated hospitalization with intensive care; $F_{i}^{H}\left(t\right)$ denotes unvaccinated hospitalization and release from ICU (intensive care unit); $V_{i}^{2}\left(t\right)$ denotes second-dose vaccinated; $V_{i}^{3}\left(t\right)$ denotes third-dose vaccinated; $V_{i}^{4}\left(t\right)$ denotes fourth-dose vaccinated; $E_{i}^{V}\left(t\right)$ denotes vaccinated and exposed; $H_{i}^{MV}\left(t\right)$ denotes vaccinated with hidden infection; $I_{i}^{V}\left(t\right)$ denotes vaccinated and infectious; $Q_{i}^{MV}\left(t\right)$ denotes vaccinated quarantined mild-symptom patient; $F_{i}^{MV}\left(t\right)$ denotes vaccinated nonquarantined mild-symptom patient; $Q_{i}^{HV}\left(t\right)$ denotes vaccinated hospitalization without intensive care; $Q_{i}^{IV}\left(t\right)$ denotes vaccinated hospitalization with intensive care; $F_{i}^{HV}\left(t\right)$ denotes vaccinated hospitalization with release from ICU; $R_{i}\left(t\right)$ denotes recovered; $S_{i}^{R}\left(t\right)$ denotes susceptible people that have been infected; $E_{i}^{R}\left(t\right)$ denotes exposed people that have been infected. A detailed description of the parameters used in the model is provided in Table 1 and the schematic diagram for the model is given in Figure 2.

**Table 1:** Parameter definitions and baseline values used in the numerical simulations.

Parameter	Description	Value	Reference
$\Lambda_{i}$	The force of infection for age group i	$\overset{8}{\underset{k=1}{\sum }}[\beta_{ik}(\left(I_{k}+I_{k}^{V}\right)+\left(H_{k}+H_{k}^{V}\right)+\theta_{F}\left(F_{k}^{M}+F_{k}^{MV}\right))/N_{k}]$	Computed
$\beta_{ik}$	Transmission rate from age group k to i	Given in Supplementary material Section 1	Estimated
$v_{2i}$	Daily second vaccination doses for age group i	*0, 241.1, 137.3, 186.6, 136.0, 92.6, 67.0, 70.6	(28)
$v_{3i}$	Daily third vaccination doses for age group i	*0, 390.3, 179.2, 232.0, 164.1, 119.8, 85.4, 82.7	(28)
$v_{4i}$	Daily fourth vaccination doses for age group i	*0, 1,398.8, 2,687.9, 2,401.4, 3,291.4, 4,659.6, 7,040.0, 9,533.8	(28)
$\tau_{2}$	Second-dose vaccine efficacy	0.06	(29)
$\tau_{3}$	Third-dose vaccine efficacy	0.39	(29)
$\tau_{4}$	Fourth-dose vaccine efficacy	0.49	(29)
ρ	Screening rate for confirmation of patients	0.7, varies	Assumed
1/α	Latent period	5.2	(30)
$\delta_{i}{}^{M}$	Probability of unvaccinated cases having mild symptoms	$1-\delta_{i}{}^{H}-\delta_{i}{}^{I}$	–
$\delta_{i}{}^{H}$	Probability of unvaccinated hospitalization without intensive care	$12.81\times \delta_{i}^{I}$	(28)
$\delta_{i}{}^{I}$	Probability of unvaccinated hospitalization with intensive care	*[0.0050, 0.0096, 0.0073, 0.0118, 0.0299, 0.0792, 0.214, 1.08] $\times 10^{-2}$	Estimated (28)
$\delta_{i}{}^{MV}$	Probability of vaccinated cases having mild symptoms	$1-\delta_{i}{}^{HV}-\delta_{i}{}^{IV}$	–
$\delta_{i}{}^{HV}$	Probability of vaccinated hospitalization without intensive care	$12.81\times \delta_{i}^{IV}$	(28)
$\delta_{i}{}^{IV}$	Probability of vaccinated hospitalization with intensive care	*[0, 0.0078, 0.0016, 0.0025 0.0338, 0.0646, 0.237, 1.366] $\times 10^{-3}$	Estimated (28)
1/q	Mean duration of case confirmation	3	(31)
$1/\gamma^{A}$	Recovery period of asymptomatic cases	3.5	(10)
$1/\gamma^{M}$	Recovery (or quarantine) period of mild-symptom cases	7	(28)
$1/\gamma_{i}^{H}$	Recovery period of hospitalization without intensive care cases	*11, 11.6, 13.2, 12.6, 13, 13.2, 14.2, 16.6	(28)
$1/\gamma_{i}{}^{I}$	Recovery period of hospitalization with intensive care cases	*15.32, 15.99, 18.66, 17.70, 17.84, 18.44, 19.77, 23.79	(28)
$1/\eta^{I}$	Period of stay in intensive care unit for critically ill patients	7	(28)
$1/\eta^{Q}$	Quarantine period	Vary (7, 5, 3, 0)	Assumed
$1/\eta^{F}$	Infectious period after release from quarantine	$1/\gamma^{M}-1/\eta^{Q}$	–
$1/\zeta^{I}$	Period of stay in the general ward for critically ill patients released from the intensive care unit	$1/\gamma^{I}-1/\eta^{I}$	–
$\kappa_{i}$	Mortality rate of unvaccinated hospitalization with intensive care cases	*0.000, 0.250,0.143, 0.333, 0.333, 0.404, 0.362, 0.490	Estimated (28)
$\kappa_{i}^{V}$	Mortality rate of vaccinated hospitalization with intensive care cases	*0, 0.250, 0.200, 0.179, 0.283, 0.367, 0.320, 0.422	Estimated (28)
$\theta_{F}$	Relative infectiousness of early release patients	0.52, when $1/\eta^{Q}=5$ 0.75, when $1/\eta^{Q}=3$ 1, when $1/\eta^{Q}=0$	(8)
1/ψ	Average duration of infectious antibodies	180	(32)

* For cells with an asterisk (*), values from left to right are for age groups: 0–9, 10–19, 20–29, 30–39,40–49, 50–59, 60–69, and 70+. The description of estimation of $\delta_{i}{}^{I}$ , $\;\delta_{i}{}^{IV}$ , $\;\kappa_{i}{}^{I}$ , and $\;\kappa_{i}{}^{I}$ is given in Supplementary material Section 1.2. In addition, we have performed the sensitivity analysis for the parameters such as β , $\tau_{2}$ , $\tau_{3}$ , $\tau_{4}$ , ρ , $\delta_{i}$ , $\delta_{i}^{V}$ , $\kappa_{i}$ , and $\kappa_{i}^{V}$ , and the result is given in Supplementary material Section 7.

**Figure 2:**
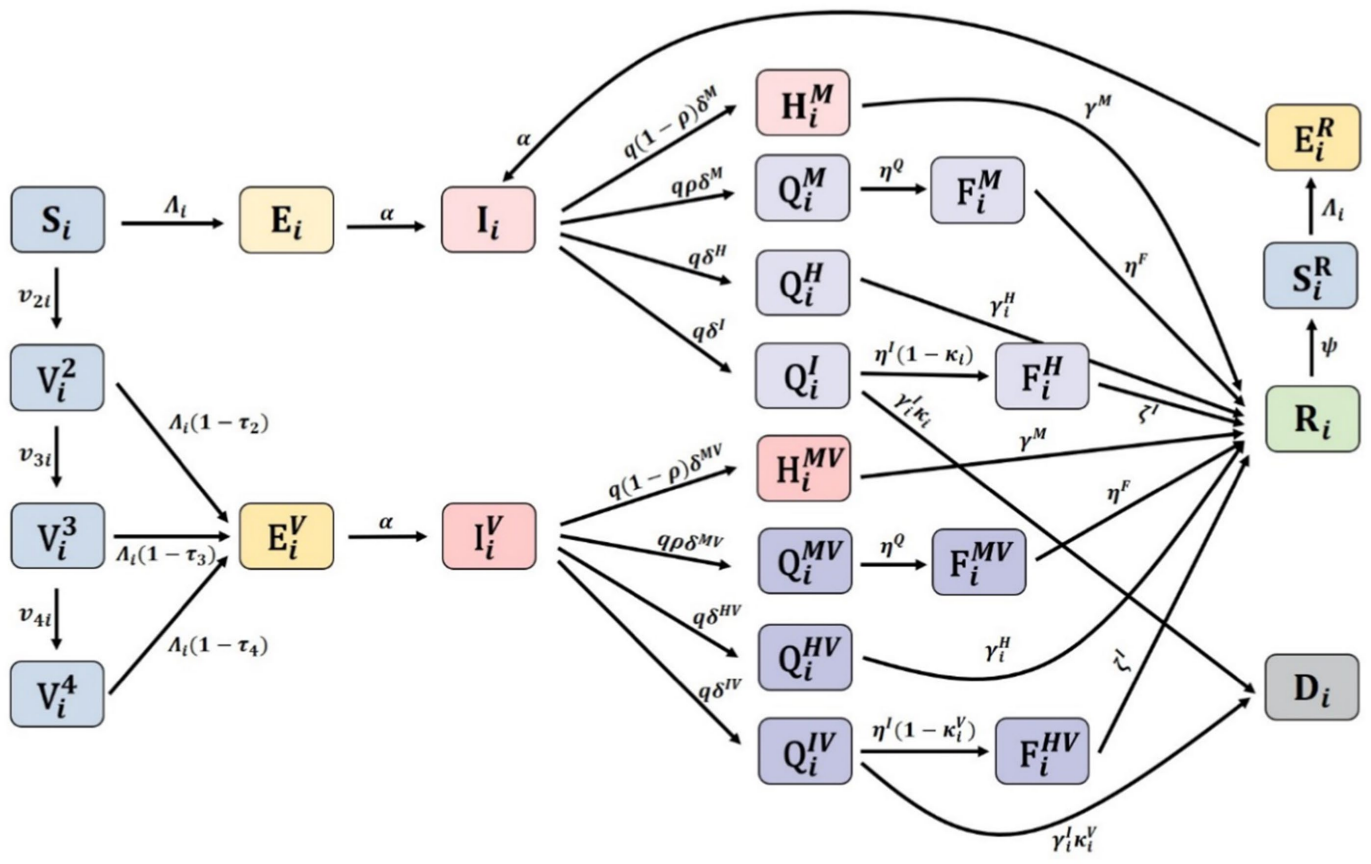
Schematic diagram of the model.

### 2.3. Parameter estimation

#### 2.3.1. Estimation of disease transmission rate

In Korea, the Omicron variant began to become the dominant variant and a pandemic wave started in February 2022. The confirmed case data by age in Korea from February 1, 2022, to December 31, 2022, were divided into four periods according to the epidemiological characteristics, and they were used to estimate the disease transmission rate. The period was divided into four intervals according to the epidemiological characteristics, and the disease transmission rate that corresponded to each interval was estimated. Each compartment of the mathematical model was divided into eight groups, and the disease transmission rate ( β ) was estimated using the least-squares method as an 8×8 matrix. The data-fitting plots and disease transmission rate matrices are provided in Supplementary material Section 1.1.

#### 2.3.2. Estimation of the transmission rate increase due to indoor mask removal

The increase in the transmission rate due to indoor mask removal was estimated according to Goyal et al. (33). Assuming that the current condition is a mask-wearing rate of 75% and a mask-wearing time of 75% (75–75%), two other scenarios were considered according to indoor mask removal: S1: 50–50%, and S2: 25–25%. For example, 50–50% means that 50% of people wear masks 50% of the time, where time is 24 h in a day (33). The increase in the disease transmission rate was calculated by comparing the values of the effective reproduction number ( $R_{t}$ ) that corresponded to the cases of 75–75%, 50–50%, and 25–25% provided by Goyal et al. (33). The disease transmission rates were increased by 17 and 35% for S1 and S2, respectively. This increased rate was evenly applied across all age groups. The derivation of the $R_{t}$ from the model is provided in Supplementary material Section 2.1, and the graph of the relationship between the $R_{t}$ and transmission rate ( β ) are provided in Supplementary material Section 2.2.

#### 2.3.3. Cost parameters

The costs due to COVID-19 include medical expenses and wage losses due to quarantine and hospitalization (22), and they were calculated as follows. The cost of the medical expenses was computed considering the average daily cost of treatment and the recovery period with respect to the statuses of the patients (i.e., mild-symptom cases, hospitalization cases without intensive care, or hospitalization cases with intensive care). The cost of the wage losses for patients older than 20 years was computed considering the average daily income, employment rate, and recovery period for each age group. The cost of the wage losses for patients younger than 19 years was computed as the income decrease for females with infected children younger than 19 years. The cost of purchasing a mask was determined by multiplying the total population by the average mask price, mask-wearing rate, and mask-wearing time. The cost computation formulae and the cost parameter table that was used are presented in Supplementary material Section 3.

## 3. Results

### 3.1. Effect of the variation in the quarantine duration

**Figure 3:**
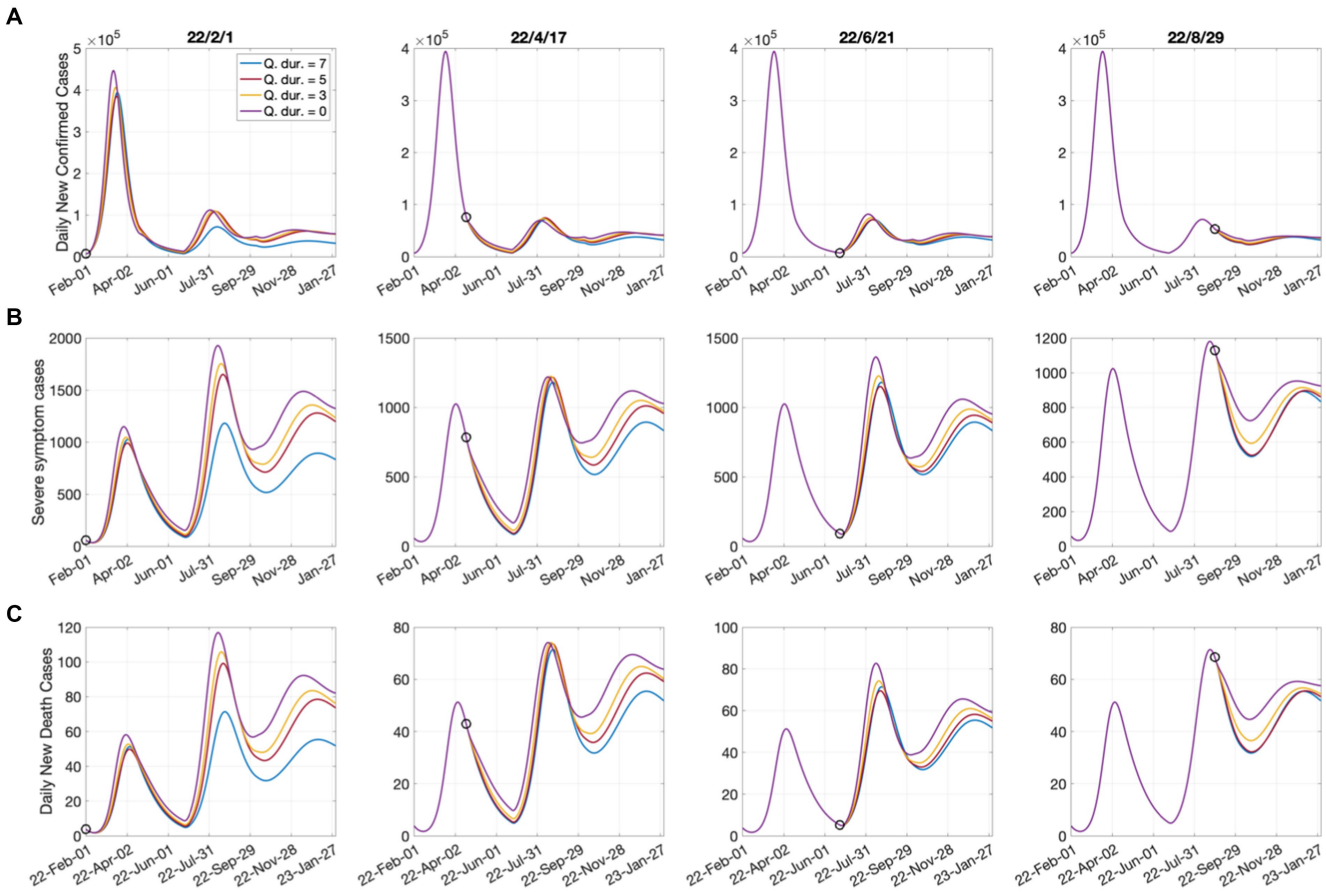
Effects of shortening the quarantine duration for each time point on **(A)** confirmed cases; **(B)** severe cases; **(C)** deaths. Open circles represent the start dates of shortening the quarantine duration.

Figure 3 shows the effect of shortening the quarantine period on the numbers of confirmed cases, critically ill patients, and deaths. The start date of shortening the quarantine period was considered according to the epidemiological characteristics, as follows:

- February 1, 2022: The Omicron epidemic began in South Korea (numerical simulation start date);
- April 17, 2022: The complete lifting of social distancing;
- June 21, 2022: The date of the lowest number of confirmed cases since the large outbreak;
- August 29, 2022: The number of critically ill patients that decreased for 7 consecutive days.

It was assumed that the quarantine period was shortened from the current 7 days to 5 days, 3 days, and 0 days. According to Figure 3, the shortening of the quarantine period had a greater impact on the increase in the numbers of critically ill patients and deaths than on the number of confirmed cases. Shortening the quarantine period to 5 or 3 days may not cause immediate increases in patient numbers; however, it could have significant long-term effects. In addition, the shortening of the quarantine period on June 21, when the number of confirmed cases was minimal in the short-term, led to more significant increases in critically ill patients and deaths compared with when it was implemented on April 17. Because June 21 can be considered the beginning of a new outbreak, shortening the quarantine period was effective when the number of confirmed cases decreased to some extent. The tables of the cumulative number of confirmed cases, number of critically ill patients, and number of deaths, as well as their increasing rates, according to the quarantine period that corresponds to each date are presented in Supplementary material Section 4.

### 3.2. Effects of changes in screening rates and activities of early releasers

**Figure 4:**
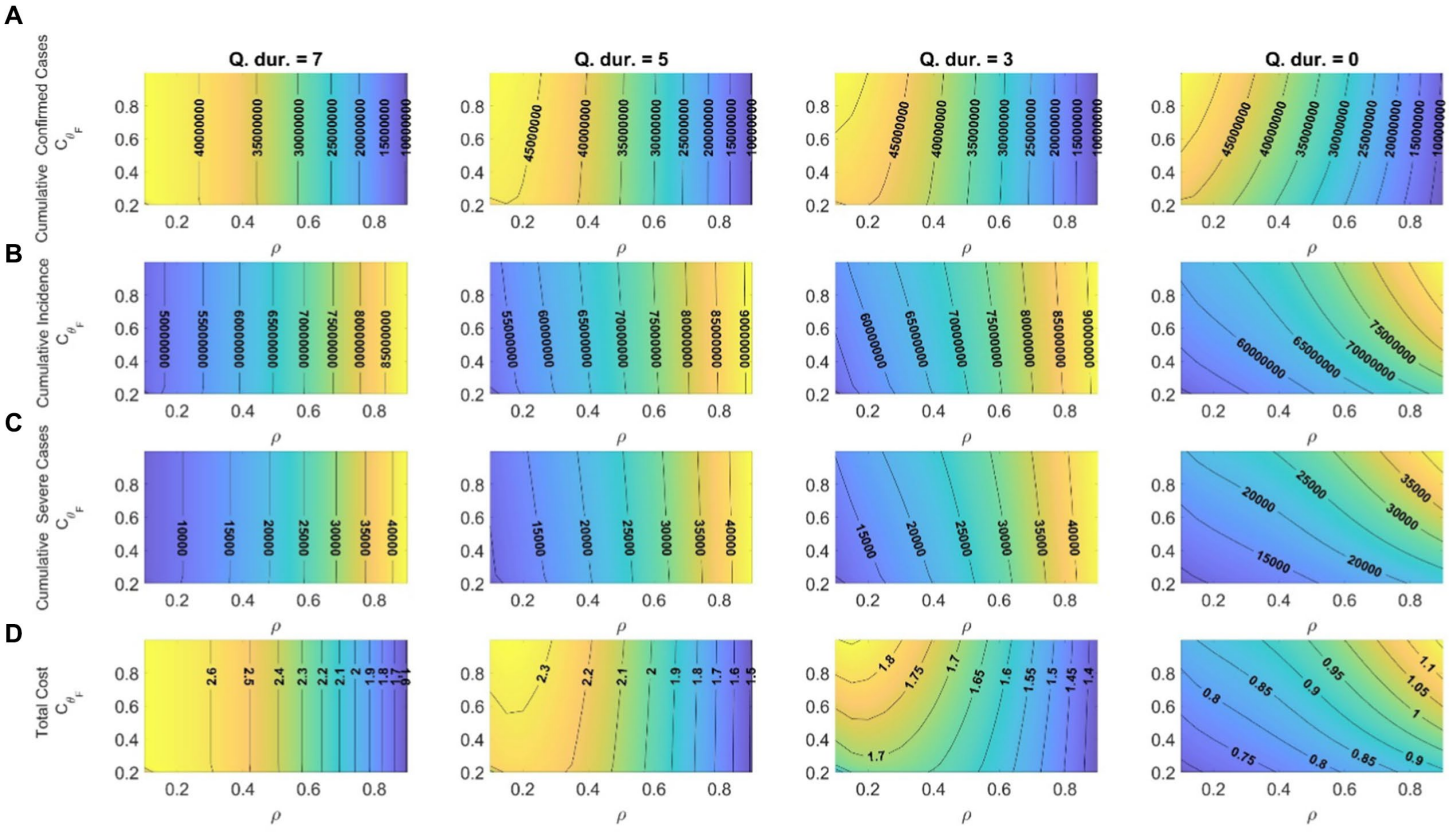
Effects of changes in screening rates and activities of early release patients on **(A)** cumulative confirmed cases; **(B)** cumulative incidence; **(C)** cumulative severe-symptom cases; **(D)** total costs ( $\times 10^{-10}\;\$$ ) corresponding to quarantine durations of 7, 5, 3, and 0 days (start date of shortening the quarantine duration: April 17, 2022).

The screening rate (*ρ*) is the proportion of mild-symptom patients who are officially confirmed and placed under quarantine, compared to the total number of such patients. A low screening rate leads to a decrease in the number of confirmed cases, but an increase in the number of infected and critically ill patients. To account for the early release of patients, we multiplied their relative infectiousness ( $\theta_{F}$ ) by the control parameter ( $C_{\theta_{F}}$ ), which varied between 0.2 and 1. Figure 4 illustrates the effects of changes in the screening rate of mild-symptom patients (*ρ*) and the activities of early releasers ( $C_{\theta_{F}}$ ) on the number of confirmed cases, incidence, severe-symptom cases, and costs losses for quarantine durations of 7, 5, 3, and 0 days starting from April 17, 2022. The values indicated by the numbers on the contour lines in the figure correspond to the number of confirmed cases, incidence, severe-symptom cases, and costs for the respective $C_{\theta_{F}}$ and *ρ* values. The results indicate that, when the mandatory quarantine duration was 5 days or longer, changes in the screening rate had a greater impact than the activity tendencies of early release patients.

Moreover, decreasing the screening rate and quarantine duration led to a reduction in wage losses caused by quarantine, which subsequently lowered the related costs. Refraining from activities of early release patients was more effective, especially with respect to cost reduction, when the mandatory quarantine period was short or nonexistent. Therefore, if the quarantine period is shortened, a campaign to encourage patients to avoid activities on their own is necessary. When the screening rate was high, there was no significant difference in costs between 5 days and 7 days quarantine periods, so it appears better to shorten the quarantine period to 3 days or less to reduce social costs. The results of implementing a shorter quarantine period on different dates are provided in Supplementary material Section 4.

### 3.3. Effects of the removal of the indoor mask mandate

South Korea was one of the countries that maintained the indoor mask mandate the longest. The removal of the mask mandate was discussed for a long time, and the decision was made to lift the indoor mask mandate in high-risk facilities on January 30, 2023, according to the criteria for doing so (see Supplementary material Section 5). The removal of masks and the reduction in the quarantine period were considered on the following break points: BP1: October 1, 2022: simulation start date; BP2: February 23, 2023:14 consecutive days of a decrease in confirmed cases; BP3: March 25, 2023: 7 consecutive days of a decrease in the number of critically ill patients.

**Figure 5:**
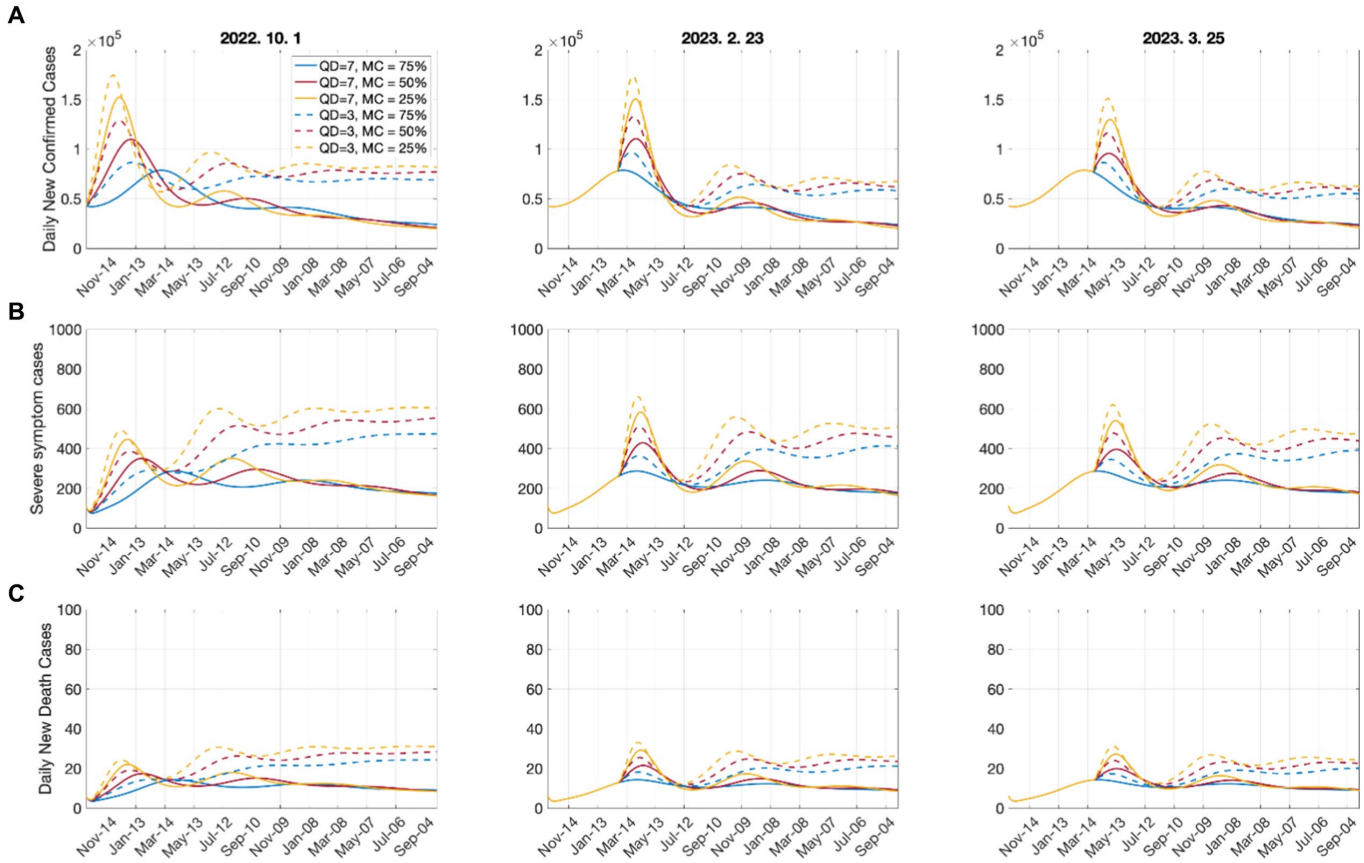
Effects of the quarantine period and mask-wearing rate on **(A)** number of confirmed cases; **(B)** number of critically ill patients; **(C)** number of deaths. QD: quarantine duration; MC: mask coverage. Dates above figures refer to days when the mandatory wearing of indoor masks was lifted.

Figure 5 shows the effect of the quarantine period and mask-wearing rate on the number of confirmed cases, critically ill patients, and deaths. The results show that the numbers of confirmed cases and critically ill patients significantly increased in the short term due to mask removal; however, in terms of the long-term perspective, the effect of the mask-wearing rate was not significant and maintaining the quarantine period was much more important. The simultaneous lifting of both the quarantine and mask wearing would result in a substantial increase in the number of critically ill patients, which requires careful consideration in decision making.

**Figure 6:**
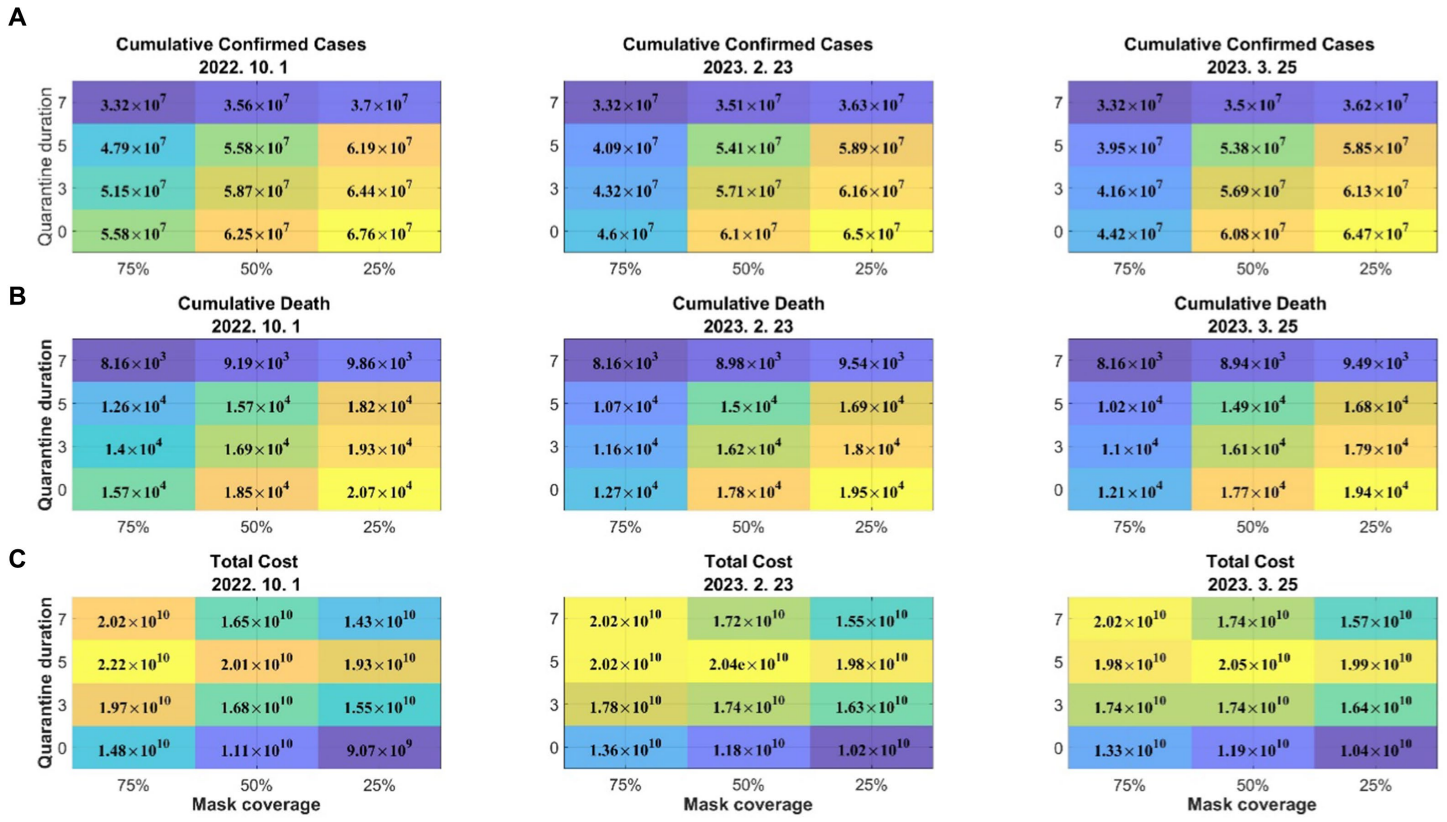
Effects of the quarantine period and mask-wearing rate on **(A)** the cumulative number of confirmed cases, **(B)** cumulative death, and **(C)** total costs. Dates above figures refer to days when the mandatory wearing of masks was lifted.

Figure 6 shows the cumulative numbers of confirmed cases and deaths and total costs, including medical expenses, wage losses, and mask costs, according to the quarantine period and mask-wearing rate for 1 year. When the quarantine period was 7 days, the increase in the number of confirmed cases and critically ill patients due to mask removal was not significant; however, the costs substantially decreased. Therefore, considering the number of confirmed cases and the costs, removal of the indoor mask mandate is efficient, but maintaining the quarantine period is necessary. The medical, wage loss, and mask costs for each scenario are provided in Supplementary material Section 6.

### 3.4. Effects of the variation in the transmission rate and vaccine efficacy

**Figure 7:**
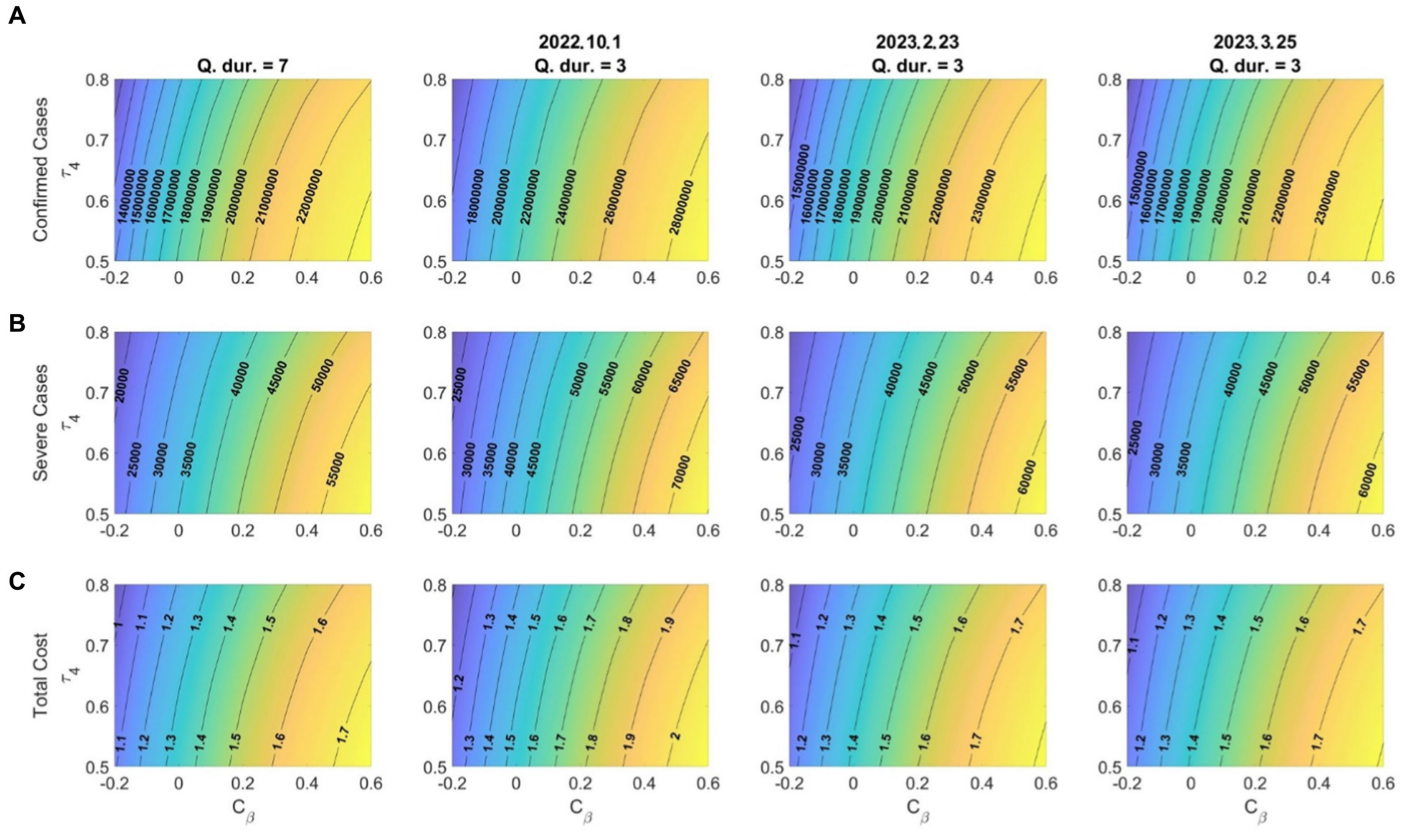
Effects of the variation in the transmission rate (C*β*)and booster vaccination efficacy $\left(\tau_{4}\right)\;$ for **(A)** confirmed cases; **(B)** severe-symptom cases; **(C)** costs due to medical expenses and wage losses ( $\times 10^{-10}$ ). Dates in titles refer to the start dates of shortened quarantine periods.

As various mutations of the SARS-CoV-2 virus occur and become prevalent, the rate of transmission of the disease may increase; however, it may decrease according to the government’s quarantine policy in response. The vaccine efficiency may increase as vaccines that respond to prevalent mutations, such as bivalent vaccines, are developed. Figure 7 illustrates the effects on the number of confirmed cases, number of severe-symptom patients, and costs related to medical expenses and wage losses according to the changes in the disease transmission rate and vaccine efficiency. The variation in the transmission rate matrix ( β ) was considered $\beta \times \left(1+C_{\beta }\right)\;$ for the control parameter ( $C_{\beta }$ ). In the simulation, the vaccination of infected persons was not considered, and the vaccination period was assumed to be 60 days. The result shows that the numbers of confirmed cases and critically ill patients were more sensitive to changes in the disease transmission rates than to changes in the vaccine efficiency. Moreover, the higher the disease transmission rate, the greater the effect of the vaccine efficiency. In other words, the more prevalent the mutations with high transmission rates, the more important vaccination with high efficiency becomes. Additionally, the results indicated that as the disease transmission rate increased, so did the impact of early quarantine release. That is, the higher the transmission rate, the greater the importance of quarantine. Although there was no clear difference, the effect of the vaccine efficiency on the number of critically ill patients and the costs was greater than that on the number of confirmed cases.

## 4. Discussion and conclusion

The infectivity of the Omicron variant of COVID-19 vanishes almost 7 days postinfection. Consequently, the mandatory quarantine period for confirmed cases in Korea has been established as 7 days. Nevertheless, the lengthy quarantine period has led to many individuals, particularly self-employed individuals, avoiding quarantine due to economic losses, and there have been discussions about shortening the quarantine period based on various social and economic considerations. Another key policy to reduce the COVID-19 infectivity is the mask-wearing policy. As of February 2023, the mandatory wearing of masks had been lifted in most countries. In the case of Korea, the mandatory wearing of masks outdoors and indoors was lifted on September 26, 2022, and on January 30, 2023, respectively.

In this study, we developed a mathematical model to study the effect of loosening the COVID-19 control measures of quarantine for confirmed cases and mandatory mask-wearing. The model parameters were estimated to incorporate vaccination and reinfection into the model. By using the mathematical model, we investigated the changes in confirmed cases, severely ill patients, and deaths over time according to different quarantine periods and mask-wearing rates. We also analyzed the impact of quarantine and mandatory mask wearing from a cost-effectiveness perspective.

One of our findings is that the increase in the number of severely ill patients due to the shortening of the mandatory quarantine period was substantially affected by the timing of the relaxation of quarantine, and the determination of the optimal time to lift the quarantine was crucial and required an analysis of the trend of confirmed cases. Moreover, the results show that shorter quarantine periods led to increases in the numbers of confirmed cases, severely ill patients, and deaths. In particular, the shorter the mandatory quarantine period for confirmed cases, the relatively higher the numbers of severely ill patients and deaths. In the case of early release from quarantine, the impact of the activities of early release patients was considered. When implementing the early quarantine release policy, it is necessary to publicize the policy requesting early release patients to voluntarily refrain from having close contact with others.

From a cost perspective, the findings of this study suggest that eliminating the quarantine policy is the most cost-effective. However, it is important to consider the potential impact on the spread of COVID-19 and to balance the need for controlling the spread of the virus with the reduction in social costs.

Our results indicate that the impact of quarantine for confirmed patients was greater than that of mandatory indoor mask-wearing. In particular, our findings reveal a sharp rise in confirmed cases and critically ill patients in the short term due to reduced mask wearing. However, the impact of mask wearing fades in the long term, which highlights the crucial role of quarantine in controlling the spread of the disease (Figure 5). Additionally, mandatory mask wearing was found to be costly and did not significantly reduce the number of confirmed cases, severely ill patients, and deaths. For optimal cost-effectiveness, the lifting of both the quarantining of confirmed cases and mandatory mask wearing is recommended, even though this would lead to a substantial increase in critically ill patients. We found that when the quarantine duration was set at 7 days, the impact of reduced mask wearing on the confirmed cases and critically ill patients was minimal, yet the costs significantly decreased (Figure 6). Thus, weighing the confirmed cases and costs, lifting mandatory indoor mask wearing is cost-effective; however, maintaining the quarantine period remains essential.

The relationship between the disease transmission rate and vaccine efficiency was also apparent, with a higher transmission rate having a greater impact on the vaccine efficiency (Figure 7), which implies that, as mutations with high transmission rates become more widespread, vaccination with high efficiency becomes increasingly important. Additionally, our findings indicate that a higher disease transmission rate amplifies the effect of early quarantine release, which means that the more widespread the disease, the more crucial quarantine becomes.

This study has two limitations that should be considered. First, the reduction in the severity of reinfections was not considered due to a lack of relevant data. Medić et al. (34) and Abu-Raddad et al. (35) have shown that the rate of severe disease is lowered with reinfection. However, Hwang et al. (36) highlights that the severity of the infection varies depending on the type of virus that the individual is initially infected with. Second, we did not account for the impact of the bivalent vaccine. As the Omicron variant has become dominant, research has shown that the efficacy of the existing vaccines against it and its subvariants has decreased. In response, a bivalent vaccine was developed. Lin et al. (37) and Surie et al. (38) demonstrated that the bivalent vaccine is highly effective as a booster vaccine against hospitalization and the severe symptoms caused by the Omicron variants. However, Kurhade et al. (39) concluded that it is too soon to determine the effectiveness of the bivalent vaccine against the Omicron variant and its subvariants, as the currently available bivalent vaccines are based on the BA.1, BA4, and BA5 variants.

In conclusion, this study developed a mathematical model to investigate the impact of loosening COVID-19 control measures, specifically quarantine for confirmed cases and mandatory mask-wearing. The results indicate that shorter quarantine periods led to increases in the numbers of confirmed cases, severely ill patients, and deaths. From a cost perspective, eliminating the quarantine policy is cost-effective, but it is important to balance the need for controlling the spread of the virus with the reduction in social costs. The study also found that mandatory mask-wearing was not cost-effective and did not significantly reduce the number of confirmed cases, severely ill patients, and deaths. The impact of quarantine for confirmed patients was found to be greater than that of mandatory mask-wearing, and the lifting of both policies is recommended for optimal cost-effectiveness, despite the increase in critically ill patients. The study also highlights the importance of considering the timing of the relaxation of quarantine and the impact of vaccine efficiency on disease transmission rates. The authors expect that this study will provide valuable insights for policymakers in balancing public health concerns and economic considerations in controlling the spread of COVID-19 or other potential pandemics in the future.

## Data availability statement

Publicly available datasets were analyzed in this study. This data can be found at: https://www.kdca.go.kr/index.es?sid=a2.

## Author contributions

JEK and CHL: conceptualization and funding acquisition. JEK, HC, and CHL: writing—original draft, review and editing, and validation. JEK, HC, and ML: formal analysis, visualization, and methodology. All authors contributed to the article and approved the submitted version.

## Funding

JEK was supported by the Korea Science Academy of KAIST with funds from the Ministry of Science and ICT. CHL was supported by a National Research Foundation of Korea (NRF) grant funded by the Korea government (MSIT) (2022R1F1A1064487), the BK21 Program (Next Generation Education Program for Mathematical Sciences, 4299990414089) funded by the Ministry of Education (MOE, Korea) and National Research Foundation of Korea (NRF), and UNIST Fundamental Science Research Grants (1.220123.0 & 1.230065.01).

## Conflict of interest

The authors declare that the research was conducted in the absence of any commercial or financial relationships that could be construed as a potential conflict of interest.

## Publisher’s note

All claims expressed in this article are solely those of the authors and do not necessarily represent those of their affiliated organizations, or those of the publisher, the editors and the reviewers. Any product that may be evaluated in this article, or claim that may be made by its manufacturer, is not guaranteed or endorsed by the publisher.

## Supplementary material

The Supplementary material for this article can be found online at: https://www.frontiersin.org/articles/10.3389/fpubh.2023.1166528/full#supplementary-material

## References

[1] Ministry of Health & Welfare. Social distancing restrictions to be lifted starting April 18. (2022) Available at: http://www.mohw.go.kr/eng/nw/nw0101vw.jsp?PAR_MENU_ID=1007&MENU_ID=100701&page=5&CONT_SEQ=371146 (Accessed February 15, 2023).

[2] Ministry of Health & Welfare. Post-omicron response plan: toward a safe, sustainable new normal. (2022) Available at: http://www.mohw.go.kr/eng/nw/nw0101vw.jsp?PAR_MENU_ID=1007&MENU_ID=100701&page=5&CONT_SEQ=371145 (Accessed February 15, 2023).

[3] Al-Tuwairqi SM Al-Harbi SK. A time-delayed model for the spread of COVID-19 with vaccination. Sci Rep. (2022) 12:19435. doi: 10.1038/s41598-022-23822-5, PMID: 36372827 PMC9659561

[4] Paul JN Mbalawata IS Mirau SS Masandawa L. Mathematical modeling of vaccination as a control measure of stress to fight COVID-19 infections. Chaos Solitons Fractals. (2023) 166:112920. doi: 10.1016/j.chaos.2022.112920, PMID: 36440088 PMC9678855

[5] Bosetti P Kiem CT Andronico A Paireau J Levy-Bruhl D Alter L. Impact of booster vaccination on the control of COVID-19 Delta wave in the context of waning immunity: application to France in the winter 2021/22. Eur Secur. (2022) 27:2101125. doi: 10.2807/1560-7917.ES.2022.27.1.2101125, PMID: 34991778 PMC8739339

[6] Ngonghala CN Taboe HB Safdar S Gumel AB. Unraveling the dynamics of the omicron and Delta variants of the 2019 coronavirus in the presence of vaccination, mask usage, and antiviral treatment: dynamics of the omicron and Delta variants of COVID-19 in the presence of control measures. Appl Math Model. (2023) 114:447–65. doi: 10.1016/j.apm.2022.09.017, PMID: 36281307 PMC9581714

[7] Gavish N Yaari R Huppert A Katriel G. Population-level implications of the Israeli booster campaign to curtail COVID-19 resurgence. Sci Transl Med. (2022) 14:eabn9836. doi: 10.1126/scitranslmed.abn9836, PMID: 35412326 PMC9012104

[8] Hay JA Kissler SM Fauver JR Mack C Tai CG Samant RM. Quantifying the impact of immune history and variant on SARS-CoV-2 viral kinetics and infection rebound: a retrospective cohort study. elife. (2022) 11:11. doi: 10.7554/eLife.81849 PMC9711520 36383192

[9] Kang M Xin H Yuan J Taslim Ali S Liang Z Zhang J. Transmission dynamics and epidemiological characteristics of SARS-CoV-2 Delta variant infections in Guangdong, China. Eur Secur. (2022) 27:27(10). doi: 10.2807/1560-7917.ES.2022.27.10.2100815, PMID: 35272744 PMC8915401

[10] Choi Y Kim JS Kim JE Choi H Lee CH. Vaccination prioritization strategies for covid-19 in Korea: a mathematical modeling approach. Int J Environ Res Public Health. (2021) 18:4240. doi: 10.3390/ijerph18084240, PMID: 33923600 PMC8073596

[11] Zhang R Wang Y Lv Z Pei S. Evaluating the impact of stay-at-home and quarantine measures on COVID-19 spread. BMC Infect Dis. (2022) 22:648–13. doi: 10.1186/s12879-022-07636-4, PMID: 35896977 PMC9326419

[12] Yu Z Keskinocak P Steimle LN Yildirim I. The impact of testing capacity and compliance with isolation on COVID-19: a mathematical modeling study. AJPM Focus. (2022) 1:100006. doi: 10.1016/j.focus.2022.100006, PMID: 36942015 PMC9119710

[13] Ashcroft P Lehtinen S Angst DC Low N Bonhoeffer S. Quantifying the impact of quarantine duration on covid-19 transmission. elife. (2021) 10:1–33. doi: 10.7554/eLife.63704, PMID: 33543709 PMC7963476

[14] Ma QX Shan H Zhang HL Li GM Yang RM Chen JM. Potential utilities of mask-wearing and instant hand hygiene for fighting SARS-CoV-2. J Med Virol. (2020) 92:1567–71. doi: 10.1002/jmv.25805, PMID: 32232986 PMC7228401

[15] Lindsley WG Blachere FM Law BF Beezhold DH Noti JD. Efficacy of face masks, neck gaiters and face shields for reducing the expulsion of simulated cough-generated aerosols. Aerosol Sci Technol. (2021) 55:449–57. doi: 10.1080/02786826.2020.1862409, PMID: 35924077 PMC9345365

[16] Motallebi S Cheung RC Mohit B Shahabi S Alishahi Tabriz A Moattari S. Modeling COVID-19 mortality across 44 countries: face covering may reduce deaths. Am J Prev Med. (2022) 62:483–91. doi: 10.1016/j.amepre.2021.09.019, PMID: 35305777 PMC8580811

[17] Shen M Zu J Fairley CK Pagán JA An L Du Z. Projected COVID-19 epidemic in the United States in the context of the effectiveness of a potential vaccine and implications for social distancing and face mask use. Vaccine. (2021) 39:2295–302. doi: 10.1016/j.vaccine.2021.02.056, PMID: 33771391 PMC7914016

[18] Reiner RC Barber RM Collins JK Zheng P Adolph C Albright J. Modeling COVID-19 scenarios for the United States. Nat Med. (2020) 27:94–105. doi: 10.1038/s41591-020-1132-9, PMID: 33097835 PMC7806509

[19] Baek YJ Lee T Cho Y Hyun JH Kim MH Sohn Y. A mathematical model of COVID-19 transmission in a tertiary hospital and assessment of the effects of different intervention strategies. PLoS One. (2020) 15:e0241169. doi: 10.1371/journal.pone.0241169, PMID: 33104736 PMC7588052

[20] Kim N Kang SJ Tak S. Reconstructing a COVID-19 outbreak within a religious group using social network analysis simulation in Korea. Epidemiol Health. (2021) 43:e2021068. doi: 10.4178/epih.e2021068, PMID: 34607404 PMC8654504

[21] Li R Liu H Fairley CK Zou Z Xie L Li X. Cost-effectiveness analysis of BNT162b2 COVID-19 booster vaccination in the United States. Int J Infect Dis. (2022) 119:87–94. doi: 10.1016/j.ijid.2022.03.029, PMID: 35338008 PMC8938315

[22] Kim JE Choi H Choi Y Lee CH. The economic impact of COVID-19 interventions: a mathematical modeling approach. Front Public Health. (2022) 10:3203. doi: 10.3389/fpubh.2022.993745, PMID: 36172208 PMC9512395

[23] Jo Y Jamieson L Edoka I Long L Silal S Pulliam JRC. Cost-effectiveness of Remdesivir and dexamethasone for COVID-19 treatment in South Africa. Open Forum Infect Dis. (2021) 8:3. doi: 10.1093/ofid/ofab040, PMID: 33732750 PMC7928624

[24] Choi KE Kim JM Rhee JE Park AK Kim EJ Yoo CK. Molecular dynamics studies on the structural stability prediction of SARS-CoV-2 variants including multiple mutants. Int J Mol Sci. (2022) 23:4956. doi: 10.3390/ijms23094956, PMID: 35563345 PMC9106056

[25] Korea Disease Control and Prevention Agency (KDCA). Announcement of COVID-19 antibody positive rate survey results. (2022) Available at: https://www.kdca.go.kr/board/board.es?mid=a20501010000&bid=0015&list_no=720760&cg_code=&act=view&nPage=1# (Accessed February 15, 2023).

[26] Hernandez-Suarez C Murillo-Zamora E. Waning immunity to SARS-CoV-2 following vaccination or infection. Front Med. (2022) 9:972083. doi: 10.3389/fmed.2022.972083 PMC9606629 36313998

[27] Korea Policy Briefing. Announcement on end of third and fourth vaccinations for COVID-19. (2022) Available at: https://www.korea.kr/news/visualNewsView.do?newsId=148909304 (Accessed February 15, 2023).

[28] Korea Disease Control and Prevention Agency (KDCA). Available at: https://www.kdca.go.kr/index.es?sid=a3 (Accessed February 15, 2023).

[29] Grewal R Kitchen SA Nguyen L Buchan SA Wilson SE Costa AP. Effectiveness of a fourth dose of COVID-19 mRNA vaccine against the omicron variant among long term care residents in Ontario, Canada: test negative design study. BMJ. (2022) 378:e071502. doi: 10.1136/bmj-2022-071502, PMID: 35793826 PMC9257064

[30] Abrams S Wambua J Santermans E Willem L Kuylen E Coletti P. Modelling the early phase of the Belgian COVID-19 epidemic using a stochastic compartmental model and studying its implied future trajectories. Epidemics. (2021) 35:100449. doi: 10.1016/j.epidem.2021.100449, PMID: 33799289 PMC7986325

[31] Choi Y Kim JS Choi H Lee H Lee CH. Assessment of social distancing for controlling COVID-19 in Korea: an age-structured modeling approach. Int J Environ Res Public Health. (2020) 17:7474. doi: 10.3390/ijerph17207474, PMID: 33066581 PMC7602130

[32] The Korea Economic Daily. 95% of the people have corona antibodies. (2022) Available at: https://www.hankyung.com/society/article/2022061403461 (Accessed February 15, 2023).

[33] Goyal A Reeves DB Thakkar N Famulare M Cardozo-Ojeda EF Mayer BT. Slight reduction in SARS-CoV-2 exposure viral load due to masking results in a significant reduction in transmission with widespread implementation. Sci Rep. (2021) 11:11838. doi: 10.1038/s41598-021-91338-5, PMID: 34088959 PMC8178300

[34] Medić S Anastassopoulou C Lozanov-Crvenković Z Vuković V Dragnić N Petrović V. Risk and severity of SARS-CoV-2 reinfections during 2020–2022 in Vojvodina, Serbia: a population-level observational study. Lancet Reg Health Eur. (2022) 20:100453. doi: 10.1016/j.lanepe.2022.100453, PMID: 35791336 PMC9246704

[35] Abu-Raddad LJ Chemaitelly H Bertollini R. Severity of SARS-CoV-2 reinfections as compared with primary infections. N Engl J Med. (2021) 385:2487–9. doi: 10.1056/NEJMc2108120, PMID: 34818474 PMC8631440

[36] Hwang MJ Hwang I Park C Park H Son T Kim JH. Evaluation of clinical severity according to primary infection variants in patients with suspected SARS-CoV-2 reinfection. Epidemiol Health. (2022) 45:e2023007. doi: 10.4178/epih.e2023007, PMID: 36596735 PMC10106545

[37] Lin DY Xu Y Gu Y Zeng D Wheeler B Young H. Effectiveness of bivalent boosters against severe omicron infection. N Engl J Med. (2023) 388:764–6. doi: 10.1056/NEJMc2215471, PMID: 36734847 PMC9933929

[38] Surie D DeCuir J Zhu Y Gaglani M Ginde AA Douin DJ. Early estimates of bivalent mRNA vaccine effectiveness in preventing COVID-19–associated hospitalization among immunocompetent adults aged ≥65 years — IVY network, 18 states, September 8–November 30, 2022. MMWR Morb Mortal Wkly Rep. (2022) 71:1625–30. doi: 10.15585/mmwr.mm715152e2, PMID: 36580424 PMC9812444

[39] Kurhade C Zou J Xia H Liu M Chang HC Ren P. Low neutralization of SARS-CoV**-**2 omicron BA.2.75.2, BQ.1.1 and XBB.1 by parental mRNA vaccine or a BA.5 bivalent booster. Nat Med. (2022) 29:344–7. doi: 10.1038/s41591-022-02162-x 36473500

