# Supplementary material for: The effect of shortening the quarantine period and lifting the indoor mask mandate on the spread of COVID-19: a mathematical modeling approach
Source: Front Public Health. 2023 Jul 21;11:1166528. doi: 10.3389/fpubh.2023.1166528 (PMC10401846; doi:10.3389/fpubh.2023.1166528)
Supplement: Supplementary file 1 [file Data_Sheet_1.docx]

Supplementary Material

# Parameter estimation

## Estimation of the disease transmission rates

We utilized the least square method to fitting the transmission rate. The least square method minimizes the sum of the squares of residual between simulated value observed by fitted parameter and real data. In our study, the 8 x 8 matrix of the transmission rate (*β*) is fitted on daily confirmed data. Using the following residual equation, we minimized the residual;

$$Residual=\left\| \sum_{i=1}^{8} \left\{ q\left( \rho\delta^{M}+\delta^{H}+\delta^{I} \right)I_{i}+q\left( \rho^{V}\delta^{MV}+\delta^{HV}+\delta^{IV} \right)I_{i}^{V} \right\}-C_{i} \right\|,$$

$$\mathrm{where}C_{i} is real confirmed data for age group i.$$

The 8x8 matrix of the transmission rate (*β*) fitted by the least square method has been used to calculate the force of infection $\Lambda$. The force of infection $\Lambda$ is an 8-dimensional vector, where each entry$\Lambda_{i}$ denotes the infection force for the respective age group $i=1,\cdots, 8$. The force of infection $\Lambda$ is calculated as follows:

$$\left[ \begin{aligned} \Lambda_{1} \\ \Lambda_{2} \\ \Lambda_{3} \\ \Lambda_{4} \\ \Lambda_{5} \\ \Lambda_{6} \\ \Lambda_{7} \\ \Lambda_{8} \end{aligned} \right]=\left[ \begin{aligned} \sum_{k=1}^{8} \beta_{1k}\left( \left( I_{k}+I_{k}^{V} \right)+\left( H_{k}+H_{k}^{V} \right){+\theta}_{F}\left( F_{k}^{M}+F_{k}^{MV} \right))/N_{k} \right) \\ \sum_{k=1}^{8} \beta_{2k}\left( \left( I_{k}+I_{k}^{V} \right)+\left( H_{k}+H_{k}^{V} \right){+\theta}_{F}\left( F_{k}^{M}+F_{k}^{MV} \right))/N_{k} \right) \\ \sum_{k=1}^{8} \beta_{3k}\left( \left( I_{k}+I_{k}^{V} \right)+\left( H_{k}+H_{k}^{V} \right){+\theta}_{F}\left( F_{k}^{M}+F_{k}^{MV} \right))/N_{k} \right) \\ \sum_{k=1}^{8} \beta_{4k}\left( \left( I_{k}+I_{k}^{V} \right)+\left( H_{k}+H_{k}^{V} \right){+\theta}_{F}\left( F_{k}^{M}+F_{k}^{MV} \right))/N_{k} \right) \\ \sum_{k=1}^{8} \beta_{5k}\left( \left( I_{k}+I_{k}^{V} \right)+\left( H_{k}+H_{k}^{V} \right){+\theta}_{F}\left( F_{k}^{M}+F_{k}^{MV} \right))/N_{k} \right) \\ \sum_{k=1}^{8} \beta_{6k}\left( \left( I_{k}+I_{k}^{V} \right)+\left( H_{k}+H_{k}^{V} \right){+\theta}_{F}\left( F_{k}^{M}+F_{k}^{MV} \right))/N_{k} \right) \\ \sum_{k=1}^{8} \beta_{7k}\left( \left( I_{k}+I_{k}^{V} \right)+\left( H_{k}+H_{k}^{V} \right){+\theta}_{F}\left( F_{k}^{M}+F_{k}^{MV} \right))/N_{k} \right) \\ \sum_{k=1}^{8} \beta_{8k}\left( \left( I_{k}+I_{k}^{V} \right)+\left( H_{k}+H_{k}^{V} \right){+\theta}_{F}\left( F_{k}^{M}+F_{k}^{MV} \right))/N_{k} \right) \end{aligned} \right].$$

Data fitting was performed based on daily data, but the simulation output was compared to weekly averages for visual clarity in Supplementary Figure 1 and Supplementary Figure 2.

Break points in fitting periods as follows;

- February 1, 2022: Numerical simulation start date, Omicron epidemic begins in South Korea
- April 17, 2022: Complete lifting of social distancing
- June 21, 2022: The date of the lowest number of confirmed cases since the big outbreak
- August 29, 2022: Number of critically ill patients decreased for 7 consecutive days


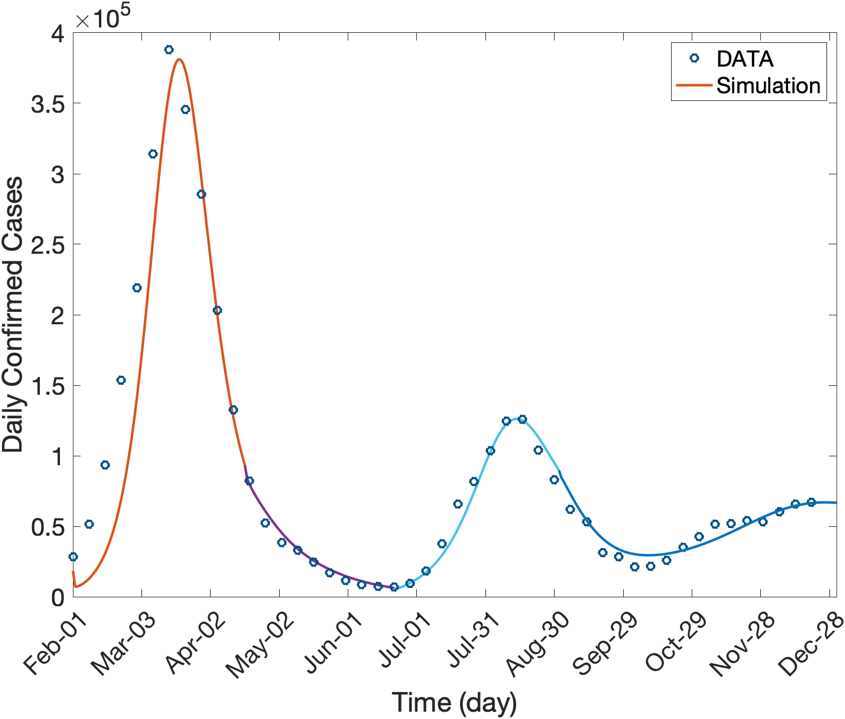


**Supplementary Figure 1.** Comparison of the confirmed case data and simulation results for daily confirmed cases of all ages from February 1, 2022 to December 31, 2022

**
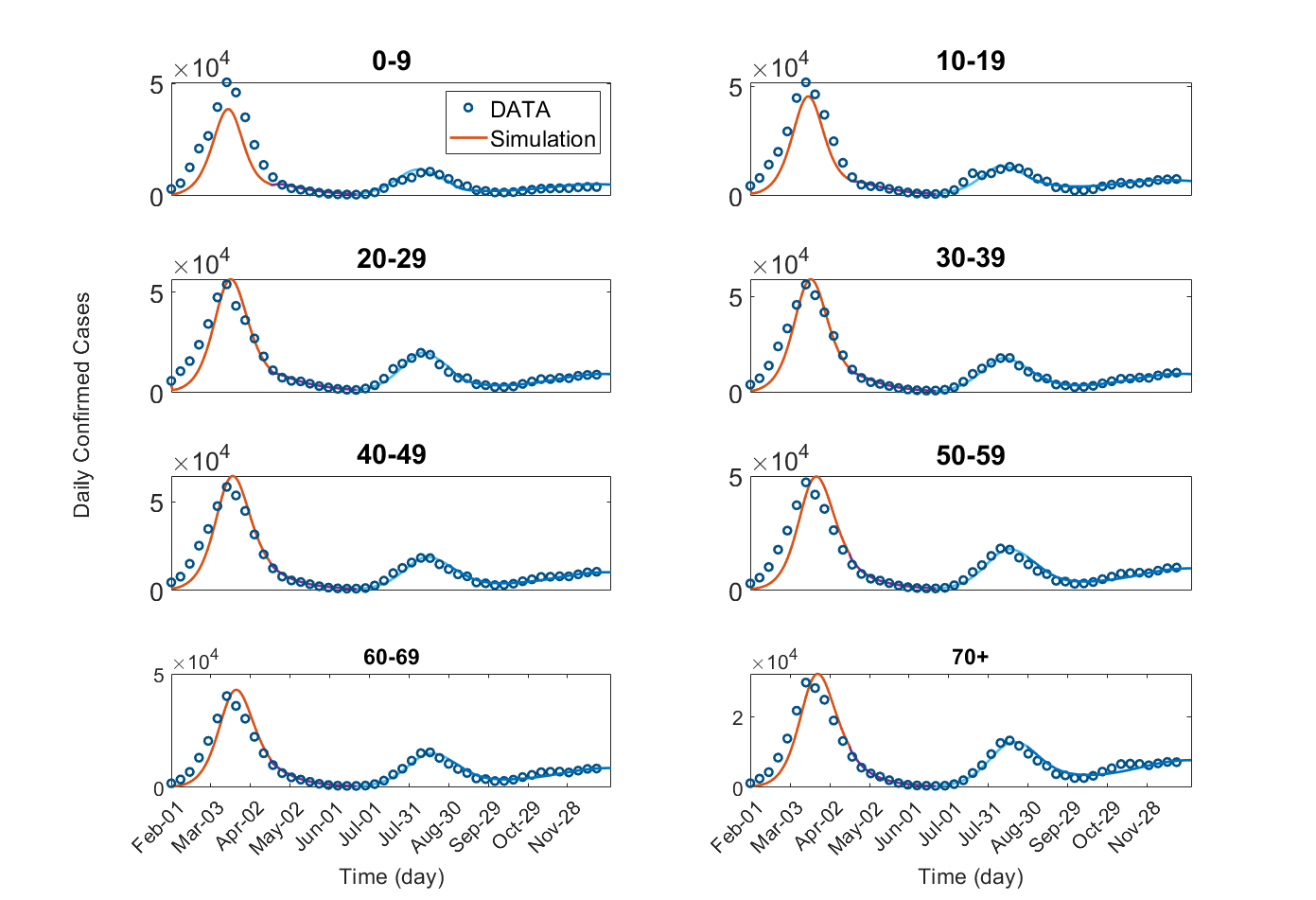
****Supplementary Figure 2.** Comparison of the confirmed case data and simulation results for daily confirmed cases of each age group from February 1, 2022 to December 31, 2022

**Supplementary Table 1.** The matrices of the transmission rate *β* for each fitting periods

**February 1, 2022 - April 16, 2022**

| 7.974E-09 | 1.415E-07 | 7.089E-07 | 3.448E-08 | 6.124E-08 | 1.258E+00 | 2.921E-10 | 1.317E-08 |
| --- | --- | --- | --- | --- | --- | --- | --- |
| 6.920E-10 | 1.804E-07 | 4.231E-07 | 1.311E-09 | 5.240E-11 | 1.506E+00 | 1.004E-11 | 5.828E-11 |
| 2.963E-07 | 8.055E-07 | 6.838E-01 | 1.107E-09 | 1.641E-07 | 9.017E-08 | 3.438E-09 | 4.425E-10 |
| 2.360E-08 | 5.930E-07 | 4.101E-01 | 8.080E-08 | 6.026E-10 | 5.807E-01 | 1.388E-08 | 7.419E-09 |
| 4.131E-08 | 7.909E-08 | 5.632E-01 | 1.238E-10 | 2.372E-12 | 2.389E-13 | 7.865E-12 | 3.015E-12 |
| 4.243E-14 | 2.620E-01 | 2.737E-09 | 3.670E-08 | 1.499E-08 | 2.305E-09 | 3.395E-10 | 7.506E-11 |
| 2.958E-01 | 4.984E-08 | 4.935E-09 | 9.609E-10 | 4.798E-10 | 2.637E-10 | 7.254E-11 | 1.556E-11 |
| 2.239E-01 | 2.348E-10 | 1.616E-11 | 7.507E-12 | 6.467E-12 | 8.957E-12 | 2.181E-12 | 4.228E-13 |

**April 17, 2022 - June 20, 2022**

| 3.055E-06 | 4.965E-06 | 3.929E-07 | 2.715E-07 | 8.751E-09 | 2.936E-10 | 8.727E-12 | 3.081E+00 |
| --- | --- | --- | --- | --- | --- | --- | --- |
| 2.155E-04 | 2.870E-04 | 1.264E-04 | 4.915E-05 | 4.233E-05 | 1.111E-05 | 5.328E-11 | 2.920E+00 |
| 4.576E-03 | 8.764E-04 | 1.632E-02 | 1.598E-01 | 4.515E-02 | 8.387E-04 | 6.000E-01 | 3.070E-01 |
| 3.766E-04 | 2.491E-03 | 3.294E-05 | 1.483E-03 | 2.618E-04 | 2.936E-06 | 3.490E-08 | 1.598E+00 |
| 9.569E-03 | 1.445E-02 | 8.513E-03 | 4.589E-05 | 1.781E-02 | 1.269E-02 | 1.571E-01 | 4.566E-01 |
| 2.630E-02 | 6.271E-03 | 3.831E-03 | 2.068E-02 | 3.593E-03 | 1.560E-03 | 1.720E-01 | 3.112E-05 |
| 4.536E-03 | 5.864E-03 | 3.973E-03 | 6.327E-03 | 5.609E-03 | 2.927E-03 | 1.125E-02 | 2.863E-01 |
| 6.978E-04 | 9.370E-04 | 6.842E-04 | 1.156E-03 | 1.640E-03 | 1.361E-03 | 1.877E-03 | 1.752E-01 |

**June 21, 2022 - August 28, 2022**

| 1.054E-01 | 8.218E-05 | 1.263E+00 | 6.351E-03 | 4.955E-03 | 1.073E-02 | 2.732E-03 | 9.568E-04 |
| --- | --- | --- | --- | --- | --- | --- | --- |
| 1.761E-01 | 8.468E-05 | 6.441E-01 | 9.776E-05 | 2.274E-03 | 1.425E-01 | 8.425E-02 | 3.409E-02 |
| 2.964E-01 | 1.762E-01 | 3.458E-01 | 1.675E-02 | 3.759E-04 | 5.172E-03 | 2.326E-03 | 6.436E-04 |
| 4.442E-01 | 1.509E-04 | 1.303E-01 | 1.018E-02 | 3.985E-04 | 1.593E-02 | 3.769E-03 | 3.269E-03 |
| 3.534E-01 | 3.443E-05 | 1.051E-05 | 4.882E-06 | 8.270E-06 | 2.534E-06 | 1.718E-08 | 2.242E-06 |
| 2.363E-01 | 4.120E-04 | 4.016E-03 | 3.687E-05 | 1.306E-04 | 6.457E-04 | 3.679E-04 | 6.746E-06 |
| 1.699E-01 | 3.618E-03 | 1.365E-02 | 6.965E-04 | 1.220E-02 | 8.706E-03 | 2.513E-04 | 4.493E-04 |
| 7.304E-02 | 7.953E-03 | 1.466E-02 | 7.519E-03 | 3.441E-03 | 2.186E-03 | 1.001E-02 | 1.666E-04 |

**August 29, 2022 - December 31, 2022**

| 4.601E-02 | 4.878E-01 | 1.609E-04 | 4.874E-06 | 2.735E-04 | 1.011E-03 | 9.219E-04 | 2.648E-03 |
| --- | --- | --- | --- | --- | --- | --- | --- |
| 2.101E-02 | 9.177E-01 | 4.106E-03 | 2.665E-03 | 4.420E-04 | 1.485E-03 | 2.409E-03 | 3.477E-03 |
| 2.900E-01 | 7.499E-02 | 1.019E-01 | 8.468E-04 | 8.954E-04 | 3.093E-02 | 6.300E-05 | 3.124E-01 |
| 5.644E-01 | 1.014E-03 | 1.883E-02 | 2.715E-05 | 5.973E-04 | 8.114E-04 | 1.322E-03 | 1.411E-04 |
| 3.521E-01 | 5.132E-04 | 1.714E-05 | 1.594E-05 | 5.094E-05 | 1.476E-05 | 1.882E-05 | 2.257E-05 |
| 2.398E-01 | 9.118E-05 | 6.740E-07 | 2.880E-05 | 1.287E-05 | 6.110E-06 | 5.505E-06 | 3.632E-06 |
| 2.025E-01 | 2.439E-03 | 6.796E-05 | 7.222E-05 | 3.553E-05 | 1.864E-05 | 1.664E-05 | 1.221E-05 |
| 7.987E-02 | 2.636E-02 | 6.076E-05 | 1.015E-06 | 1.318E-05 | 5.830E-06 | 5.535E-06 | 5.272E-06 |

## Estimation of probability of hospitalization with intensive care and mortality rate

We estimated the probability of hospitalization with intensive care and mortality rate based on the actual data provided by Korea Disease Control and Prevention Agency (KDCA) (1,2,3). The actual data contain the number of confirmed cases, severe cases, death according to their vaccination history and age groups. A detailed description for the estimation of the parameters is given as follows.

For each age group $i=1, \cdots, 8$*,* $P_{0}$*,* $P_{0}^{I}$*,* and $P_{0}^{D}$ denote the number of unvaccinated confirmed cases, the number of unvaccinated patients with intensive care, andthe number of unvaccinated death, respectively. Moreover, $P_{j}, P_{j}^{I}, \mathrm{and} P_{j}^{D}, j=1,2,3,4$ represent the number of $j$th vaccinated confirmed cases, the number of $j$th vaccinated patients with intensive care, and the number of death among $j$th vaccinated patients, respectively. The probability of unvaccinated/vaccinated hospitalization with intensive care is calculated by following equation:

$$\delta_{i}=\left( \frac{P_{0}^{I}+P_{1}^{I}}{(P_{0}+P_{1})/\rho} \right), \delta_{i}^{V}=\left( \frac{P_{2}^{I}+P_{3}^{I}+P_{4}^{I}}{(P_{2}+P_{3}+P_{4})/\rho} \right).$$

The mortality rate of unvaccinated/vaccinated hospitalization with intensive care cases is calculated by the following equation:

$$\kappa_{i}=\left( \frac{P_{0}^{D}+P_{1}^{D}}{(P_{0}+P_{1})/\rho} \right), \kappa_{i}^{V}=\left( \frac{P_{2}^{D}+P_{3}^{D}+P_{4}^{D}}{(P_{2}+P_{3}+P_{4})/\rho} \right).$$

# The effective reproduction numbers

## The derivation of the effective reproduction numbers

We computed the effective reproduction number $R_{t}$, which measures the mean number of the secondary cases infected by an infectious individual at time $t$, which is obtained by calculating the spectral radius of the next-generation matrix.

Let $x=\left( E_{i},E_{i}^{V},E_{i}^{R},I_{i},I_{i}^{V},H_{i}^{M},H_{i}^{MV},F_{i}^{M},F_{i}^{MV} \right)^{T}$ for $i=1, \cdots, 8$. Let $F\left( x \right)$ represents all of the new infections and the net transition rates of the corresponding compartments are represented by $V\left( x \right).$

$$F\left( x \right)=\left( \begin{aligned} {\Lambda_{i}S}_{i} \\ \Lambda_{i}\left( \left( 1-\tau_{2} \right)V_{i}^{2}+\left( 1-\tau_{3} \right)V_{i}^{3}+\left( 1-\tau_{4} \right)V_{i}^{4} \right) \\ {\Lambda_{i}S}_{i}^{R} \\ 0 \\ 0 \\ 0 \\ 0 \\ 0 \\ 0 \end{aligned} \right)$$

where

$$\Lambda_{i}=\sum_{k=1}^{8} \frac{\beta_{ik}(\left( I_{k}+I_{k}^{V} \right)+(H_{k}+H_{k}^{V})+\theta_{F}(F_{k}^{M}+H_{k}^{MV}))}{N_{k}}.$$

$$V\left( x \right)=\left( \begin{aligned} \alpha E_{i} \\ \alpha E_{i}^{V} \\ \alpha E_{i}^{R} \\ -\alpha\left( E_{i}+E_{i}^{R} \right)+qI_{i} \\ -\alpha E_{i}^{V}+qI_{i}^{V} \\ -q\rho\delta_{i}^{M}I_{i}+\gamma^{M}H_{i}^{M} \\ -q\rho\delta_{i}^{MV}I_{i}^{V}+\gamma^{M}H_{i}^{MV} \\ -\eta^{Q}Q_{i}^{M}+\eta^{F}F_{i}^{M} \\ -\eta^{Q}Q_{i}^{MV}+\eta^{F}F_{i}^{MV} \end{aligned} \right).$$

Then, $\mathbf{F}$ and $\mathbf{V}$ are $72*72$ matrices given by $\mathbf{F}=\left[ \frac{\partial F_{i}}{\partial x_{j}}(x_{0}) \right]$ and $\mathbf{V}=\left[ \frac{\partial V_{i}}{\partial x_{j}}(x_{0}) \right]$ with $1\leq i, j\leq72$ as a part of next generation operator where $x_{0}$ is the disease-free state. Thus, we obtain

$$\mathbf{F}\boldsymbol{=}\left[ \begin{matrix} 0_{8,8} & 0_{8,8} & 0_{8,8} & M_{A} & M_{A} & M_{A} & M_{A} & \theta_{F}M_{A} & \theta_{F}M_{A} \\ 0_{8,8} & 0_{8,8} & 0_{8,8} & M_{B} & M_{B} & M_{B} & M_{B} & \theta_{F}M_{B} & \theta_{F}M_{B} \\ 0_{8,8} & 0_{8,8} & 0_{8,8} & M_{C} & M_{C} & M_{C} & M_{C} & \theta_{F}M_{C} & \theta_{F}M_{C} \\ 0_{8,8} & 0_{8,8} & 0_{8,8} & 0_{8,8} & 0_{8,8} & 0_{8,8} & 0_{8,8} & 0_{8,8} & 0_{8,8} \\ 0_{8,8} & 0_{8,8} & 0_{8,8} & 0_{8,8} & 0_{8,8} & 0_{8,8} & 0_{8,8} & 0_{8,8} & 0_{8,8} \\ 0_{8,8} & 0_{8,8} & 0_{8,8} & 0_{8,8} & 0_{8,8} & 0_{8,8} & 0_{8,8} & 0_{8,8} & 0_{8,8} \\ 0_{8,8} & 0_{8,8} & 0_{8,8} & 0_{8,8} & 0_{8,8} & 0_{8,8} & 0_{8,8} & 0_{8,8} & 0_{8,8} \\ 0_{8,8} & 0_{8,8} & 0_{8,8} & 0_{8,8} & 0_{8,8} & 0_{8,8} & 0_{8,8} & 0_{8,8} & 0_{8,8} \\ 0_{8,8} & 0_{8,8} & 0_{8,8} & 0_{8,8} & 0_{8,8} & 0_{8,8} & 0_{8,8} & 0_{8,8} & 0_{8,8} \end{matrix} \right].$$

Here $M_{A}, M_{B},M_{C}$ is the matrix computed as

$$M_{A}=diag\{S_{1},S_{2}, \cdots,S_{8}{\}}_{8}*B*diag\{\frac{1}{N_{1}},\frac{1}{N_{2}},\cdots,\frac{1}{N_{8}}{\}}_{8}$$

$$M_{B}=Vacc*B*diag\{\frac{1}{N_{1}},\frac{1}{N_{2}},\cdots,\frac{1}{N_{8}}{\}}_{8}$$

$$M_{C}=diag\{S_{1}^{R},S_{2}^{R}, \cdots,S_{8}^{R}{\}}_{8}*B*diag\{\frac{1}{N_{1}},\frac{1}{N_{2}},\cdots,\frac{1}{N_{8}}{\}}_{8}$$

where $B=[\beta_{ij}]$, $S_{i}$ is the susceptible population of age group $i$, $V_{i}$ is the vaccinated population of age group $i$, $diag\{{\}}_{n}$ denotes the diagonal matrix with $n$ diagonal entries, and

$Vacc=diag\{{\left( 1-\tau_{2} \right)V_{1}^{2}+\left( 1-\tau_{3} \right)V_{1}^{3}+\left( 1-\tau_{4} \right)V_{1}^{4}, \cdots,\left( 1-\tau_{2} \right)V_{8}^{2}+\left( 1-\tau_{3} \right)V_{8}^{3}+\left( 1-\tau_{4} \right)V_{8}^{4} \}}_{8}$.

$$\mathbf{V}\boldsymbol{=}\left[ \begin{matrix} M_{D} & 0_{8,8} & 0_{8,8} & 0_{8,8} & 0_{8,8} & 0_{8,8} & 0_{8,8} & 0_{8,8} & 0_{8,8} \\ 0_{8,8} & M_{D} & 0_{8,8} & 0_{8,8} & 0_{8,8} & 0_{8,8} & 0_{8,8} & 0_{8,8} & 0_{8,8} \\ 0_{8,8} & 0_{8,8} & M_{D} & 0_{8,8} & 0_{8,8} & 0_{8,8} & 0_{8,8} & 0_{8,8} & 0_{8,8} \\ -M_{D} & 0_{8,8} & -M_{D} & M_{E} & 0_{8,8} & 0_{8,8} & 0_{8,8} & 0_{8,8} & 0_{8,8} \\ 0_{8,8} & {-M}_{D} & 0_{8,8} & 0_{8,8} & M_{E} & 0_{8,8} & 0_{8,8} & 0_{8,8} & 0_{8,8} \\ 0_{8,8} & 0_{8,8} & 0_{8,8} & {-\rho M}_{F} & 0_{8,8} & M_{G} & 0_{8,8} & 0_{8,8} & 0_{8,8} \\ 0_{8,8} & 0_{8,8} & 0_{8,8} & 0_{8,8} & -{\rho M}_{H} & 0_{8,8} & M_{G} & 0_{8,8} & 0_{8,8} \\ 0_{8,8} & 0_{8,8} & 0_{8,8} & 0_{8,8} & 0_{8,8} & 0_{8,8} & 0_{8,8} & M_{I} & 0_{8,8} \\ 0_{8,8} & 0_{8,8} & 0_{8,8} & 0_{8,8} & 0_{8,8} & 0_{8,8} & 0_{8,8} & 0_{8,8} & M_{I} \end{matrix} \right]$$

where $M_{D}=\alpha*\mathbf{I}_{8}$,$M_{E}=q*\mathbf{I}_{8}$, $M_{F}=q*diag\{\delta_{1}^{M},\delta_{2}^{M}, \cdots,\delta_{8}^{M}{\}}_{8}{*\mathbf{I}}_{8}{, M}_{G}=\gamma^{M}{*\mathbf{I}}_{8}$, ${, M}_{H}=q*diag\{\delta_{1}^{MV},\delta_{2}^{MV}, \cdots,\delta_{8}^{MV}{\}}_{8}{*\mathbf{I}}_{8}$, $M_{I}=\eta^{F}*\mathbf{I}_{8}$and $\mathbf{I}_{8}$ is the size 8 identity matrix.

Then, the inverse matrix of $\mathbf{V}$ is$\mathbf{V}^{\boldsymbol{-1}}\boldsymbol{=}\left[ \begin{matrix} {M_{D}}^{-1} & 0_{8,8} & 0_{8,8} & 0_{8,8} & 0_{8,8} & 0_{8,8} & 0_{8,8} & 0_{8,8} & 0_{8,8} \\ 0_{8,8} & {M_{D}}^{-1} & 0_{8,8} & 0_{8,8} & 0_{8,8} & 0_{8,8} & 0_{8,8} & 0_{8,8} & 0_{8,8} \\ 0_{8,8} & 0_{8,8} & {M_{D}}^{-1} & 0_{8,8} & 0_{8,8} & 0_{8,8} & 0_{8,8} & 0_{8,8} & 0_{8,8} \\ {M_{E}}^{-1} & 0_{8,8} & {M_{E}}^{-1} & {M_{E}}^{-1} & 0_{8,8} & 0_{8,8} & 0_{8,8} & 0_{8,8} & 0_{8,8} \\ 0_{8,8} & {M_{E}}^{-1} & 0_{8,8} & 0_{8,8} & {M_{E}}^{-1} & 0_{8,8} & 0_{8,8} & 0_{8,8} & 0_{8,8} \\ {\frac{\rho}{q\gamma^{M}}M}_{F} & 0_{8,8} & {\frac{\rho}{q\gamma^{M}}M}_{F} & {\frac{\rho}{q\gamma^{M}}M}_{F} & 0_{8,8} & {M_{G}}^{-1} & 0_{8,8} & 0_{8,8} & 0_{8,8} \\ 0_{8,8} & {\frac{\rho}{q\gamma^{M}}M}_{H} & 0_{8,8} & 0_{8,8} & {\frac{\rho}{q\gamma^{M}}M}_{H} & 0_{8,8} & {M_{G}}^{-1} & 0_{8,8} & 0_{8,8} \\ 0_{8,8} & 0_{8,8} & 0_{8,8} & 0_{8,8} & 0_{8,8} & 0_{8,8} & 0_{8,8} & {M_{I}}^{-1} & 0_{8,8} \\ 0_{8,8} & 0_{8,8} & 0_{8,8} & 0_{8,8} & 0_{8,8} & 0_{8,8} & 0_{8,8} & 0_{8,8} & {M_{I}}^{-1} \end{matrix} \right]$.

Hence, one can obtain the next generation matrix $\mathbf{G}$ as

$\mathbf{G}\boldsymbol{=}\mathbf{F}\mathbf{V}^{\boldsymbol{-1}}\boldsymbol{=}\left[ \begin{matrix} {\frac{1}{q}M_{A}+\frac{\rho}{q\gamma^{M}}M_{A}M}_{F} & {\frac{1}{q}M_{A}+\frac{\rho}{q\gamma^{M}}M_{A}M}_{H} & {\frac{1}{q}M_{A}+\frac{\rho}{q\gamma^{M}}M_{A}M}_{F} & {\frac{1}{q}M_{A}+\frac{\rho}{q\gamma^{M}}M_{A}M}_{F} & {\frac{1}{q}M_{A}+\frac{\rho}{q\gamma^{M}}M_{A}M}_{H} & \frac{1}{\gamma^{M}}M_{A} & \frac{1}{\gamma^{M}}M_{A} & \frac{\theta_{F}}{\eta_{F}}M_{A} & \frac{\theta_{F}}{\eta_{F}}M_{A} \\ {\frac{1}{q}M_{B}+\frac{\rho}{q\gamma^{M}}M_{B}M}_{F} & {\frac{1}{q}M_{B}+\frac{\rho}{q\gamma^{M}}M_{B}M}_{H} & {\frac{1}{q}M_{B}+\frac{\rho}{q\gamma^{M}}M_{B}M}_{F} & {\frac{1}{q}M_{B}+\frac{\rho}{q\gamma^{M}}M_{B}M}_{F} & {\frac{1}{q}M_{B}+\frac{\rho}{q\gamma^{M}}M_{B}M}_{H} & \frac{1}{\gamma^{M}}M_{B} & \frac{1}{\gamma^{M}}M_{B} & \frac{\theta_{F}}{\eta_{F}}M_{B} & \frac{\theta_{F}}{\eta_{F}}M_{B} \\ {\frac{1}{q}M_{C}+\frac{\rho}{q\gamma^{M}}M_{C}M}_{F} & {\frac{1}{q}M_{C}+\frac{\rho}{q\gamma^{M}}M_{C}M}_{H} & {\frac{1}{q}M_{C}+\frac{\rho}{q\gamma^{M}}M_{C}M}_{F} & {\frac{1}{q}M_{C}+\frac{\rho}{q\gamma^{M}}M_{C}M}_{F} & {\frac{1}{q}M_{C}+\frac{\rho}{q\gamma^{M}}M_{C}M}_{H} & \frac{1}{\gamma^{M}}M_{C} & \frac{1}{\gamma^{M}}M_{C} & \frac{\theta_{F}}{\eta_{F}}M_{C} & \frac{\theta_{F}}{\eta_{F}}M_{C} \\ 0_{8,8} & 0_{8,8} & 0_{8,8} & 0_{8,8} & 0_{8,8} & 0_{8,8} & 0_{8,8} & 0_{8,8} & 0_{8,8} \\ 0_{8,8} & 0_{8,8} & 0_{8,8} & 0_{8,8} & 0_{8,8} & 0_{8,8} & 0_{8,8} & 0_{8,8} & 0_{8,8} \\ 0_{8,8} & 0_{8,8} & 0_{8,8} & 0_{8,8} & 0_{8,8} & 0_{8,8} & 0_{8,8} & 0_{8,8} & 0_{8,8} \\ 0_{8,8} & 0_{8,8} & 0_{8,8} & 0_{8,8} & 0_{8,8} & 0_{8,8} & 0_{8,8} & 0_{8,8} & 0_{8,8} \\ 0_{8,8} & 0_{8,8} & 0_{8,8} & 0_{8,8} & 0_{8,8} & 0_{8,8} & 0_{8,8} & 0_{8,8} & 0_{8,8} \\ 0_{8,8} & 0_{8,8} & 0_{8,8} & 0_{8,8} & 0_{8,8} & 0_{8,8} & 0_{8,8} & 0_{8,8} & 0_{8,8} \end{matrix} \right]$.

Finally, the effective reproduction number $R_{t}$ is computed as the spectral radius $\boldsymbol{\rho}(\mathbf{G})$ of the next generation matrix $\mathbf{G}$, i.e., $R_{t}=\boldsymbol{\rho}(\mathbf{G})$.

$$R_{t}=\boldsymbol{\rho}\left( \mathbf{G} \right)=\left| \frac{\gamma^{M}\boldsymbol{(\rho}(M_{A})+\boldsymbol{\rho}(M_{B})+{\boldsymbol{\rho}(M}_{C}))+{\rho(\boldsymbol{\rho}(M}_{A}M_{F}){+\boldsymbol{\rho}(M}_{C}M_{F})+{\boldsymbol{\rho}(M}_{B}M_{H}))}{q\gamma^{M}} \right|$$

.

## Estimation of transmission rate increase due to indoor mask removal

In (4), the effective reproduction number ($R_{t}$) values are provided when the mask wearing rate and mask wearing time are 75–75%, 50–50%, and 25–25% in the Figure 3. It is shown that $R_{t}$ values are 1.1, 1.3, and 1.5 for the cases of 75–75%, 50–50%, and 25–25% of the mask wearing rate - the mask wearing time, respectively. We assumed that the mask wearing rate and mask wearing time were 75–75% on September 1, 2023, when outdoor masks were not mandatory and only indoor masks were mandatory. As of September 1, 2023, $R_{t}$was calculated to be approximately 1.1. The reproduction number formula is given in the next section. The variation in the transmission rate matrix ($\beta$) was considered $\beta\times\left( 1+C_{\beta} \right)$for the control parameter ($C_{\beta}$). It is shown that when $C_{\beta}$is 1.17 and 1.35, $R_{t}$ values are 1.3 and 1.5 respectively. The value of $R_{t}$ according to $C_{\beta}$ is shown in Supplementary Figure 2. We assumed two scenarios in which the mask wearing rate and mask wearing time decreased to 50–50% and 25–25%, respectively, when the mandatory wearing of indoor masks was lifted, and the disease transmission rates are computed by reflecting the corresponding $C_{\beta}$.


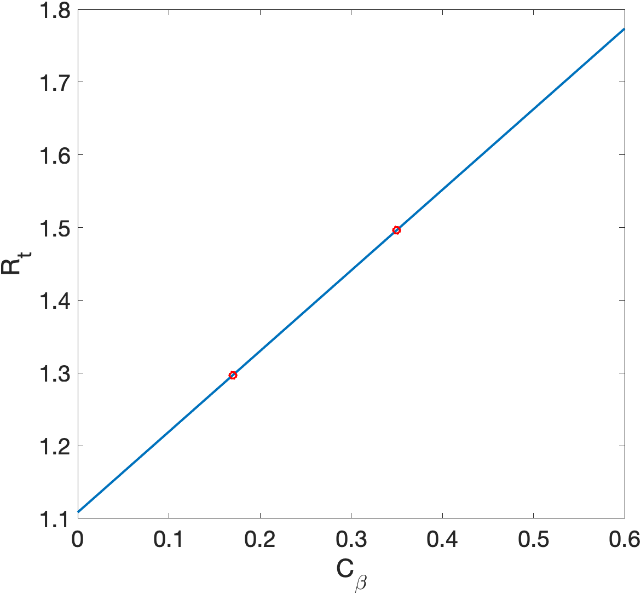


**Supplementary Figure 3.** Plot of $R_{t}$ vs. $C_{\beta}$. The red circle represents the case where $R_{t}$ values are 1.3 and 1.5, corresponding to the mask wearing rate and mask wearing time of 50–50% and 25–25%.

# Estimation of the cost due to COVID-19.

The formula for calculating the cost of COVID-19 and the parameters used are given below.

**Supplementary Table 2.** Formulae for the cost estimation

| **Medical expenses** |  |
| --- | --- |
| Mild symptom case ($H^{M}$) | $\sum_{i=1}^{8} [$ Average daily cost of treatment for $mild patients$ $\left( C_{M} \right)$ $\times$ Recovery period of mild symptom cases $\left( 1/\gamma_{i}^{M} \right)\times$ Number of mild patients $\left( Q_{i}^{M}+Q_{i}^{MV} \right)$] |
| Hospitalized case without intensive care ($H^{H}$) | $\sum_{i=1}^{8} [$ Average daily cost of treatment for hospitalized patients without intensive care $\left( C_{H} \right)$ $\times$ Recovery period of hospitalized patients without intensive care $\left( 1/\gamma_{i}^{H} \right)\times$ Number of hospitalized patients without intensive care $\left( Q_{i}^{H}+Q_{i}^{HV} \right)$] |
| Hospitalized case with intensive care ($H^{I}$) | $\sum_{i=1}^{8} [$ Average daily cost of treatment for hospitalized patients with intensive care $\left( C_{I} \right)$ $\times$ Period of stay in intensive care unit $\left( 1/{\eta^{I}} \right)\times$ Number of hospitalized patients in intensive care unit $\left( Q_{i}^{I}+Q_{i}^{IV} \right)$] +$\sum_{i=1}^{8} [$ Average daily cost of treatment for hospitalized patients without intensive care $\left( C_{H} \right)$ $\times$ Period of stay in general hospital room for critically ill patients $\left( 1/{\zeta^{I}} \right)\times$ Number of hospitalized patients released from intensive care $\left( F_{i}^{H}+F_{i}^{HV} \right)$*]* |
| **Wage loss** |  |
| Older than 20 years | $\sum_{x=M,H,I} [\sum_{i=1}^{8} [$ Average daily income in age group $i$ $\left( W_{i} \right)$ $\times$ Employment rate in age group $i$ $\left( E_{i} \right)$ $\times$Recovery period of cases $\left( 1/\gamma_{i}^{x}, 1/{\eta^{I}} or 1/{\zeta^{I}} \right)$ $\times$ Number of patients $\left( Q_{i}^{x}, Q_{i}^{xV}, F_{i}^{H}, or F_{i}^{HV} \right)$]] |
| Younger than 19 years | Average daily income of women in their 30s and 40s $\left( W_{f} \right)$ $\times$ Female employment rate of children younger than 19 years$\left( E_{f} \right)$ $\times$ Average recovery period $\times$ Number of patients younger than 19 years |
| **Mask cost** | Average mask price (MP) $\times mask wearing rate (\mu_{r})\times$mask wearing time ( $\mu_{c}$) |

**Supplementary Table 3.** Descriptions and values of parameters for cost estimation

|  | **Description** | **Value** | **Reference** |
| --- | --- | --- | --- |
| $C_{M}$ | Medical cost for mild patients per day | $5 | (5,6) |
| $C_{H}$ | Medical cost for number of hospitalized patients without intensive per day | $432.5 | (5,6) |
| $C_{I}$ | Medical cost for hospitalized patients with intensive per day | $1129.5 | (5,6) |
| $E_{i}$ | Employment rate in age group $i$ | 0, 04, 0.557, 0.753, 0.771, 0.743, 0.566, 0.23 | (7) |
| $E_{f}$ | Female employment rate with children younger than 19 years | 0.555 | (7) |
| $W_{i}$ | Average daily income in age group $i$ | 0, 55.47, 76.63, 109.40, 129.33, 127.14, 82.14, 68.08 | (8) |
| $W_{f}$ | Average daily income of women in 30s and 40s | 99.3500 | (8) |
| $g$ | Average annual salary increase rate | 0.02 | (9) |
| $r$ | Social discount rate | 0.04 | (9) |
| $N_{i}$ | Average working period in age group $i$ | 50, 50, 45, 35, 25, 15, 5, 0 | Assumed |
| MP | Average mask price | 0.4 $ | (10) |
| $\mu_{r}$ | Mask wearing rate | $0.75, 0.5, 0.25$ | (4) |
| $\mu_{c}$ | Mask wearing time | $0.75, 0.5, 0.25$ | (4) |

# Effect of variation of quarantine duration

**Supplementary Table 4.** Cumulative number of confirmed cases, number of critically ill patients, and number of deaths since the date of isolation relaxation due to reduction in quarantine period (simulation period: from the specified date to January 31, 2023)

| Date | Quarantine duration | Confirmed Cases | Severe Cases | Death |
| --- | --- | --- | --- | --- |
| 2022/02/01 | 7 | 2.31E+07 | 30120 | 11978 |
|  | 5 | 2.75E+07 | 39439 | 15711 |
|  | 3 | 2.82E+07 | 42526 | 17039 |
|  | 0 | 2.96E+07 | 48624 | 19669 |
| 2022/04/17 | 7 | 9.52E+06 | 24193 | 10245 |
|  | 5 | 1.06E+07 | 26638 | 11209 |
|  | 3 | 1.10E+07 | 28256 | 11901 |
|  | 0 | 1.17E+07 | 31430 | 13256 |
| 2022/06/21 | 7 | 7.78E+06 | 21729 | 8992.2 |
|  | 5 | 8.31E+06 | 22540 | 9288 |
|  | 3 | 8.62E+06 | 23910 | 9875.2 |
|  | 0 | 9.20E+06 | 26538 | 11001 |
| 2022/08/29 | 7 | 4.82E+06 | 14969 | 6598.4 |
|  | 5 | 5.02E+06 | 15327 | 6728.9 |
|  | 3 | 5.25E+06 | 16338 | 7155.5 |
|  | 0 | 5.68E+06 | 18305 | 7987.3 |

**Supplementary Table 5.** The rate of increase in the cumulative number of confirmed cases, the number of critically ill patients, and the number of deaths since the date of quarantine relaxation due to the reduction of the quarantine period (simulation period: from the specified date to January 31, 2023)

| Date | Quarantine duration | Confirmed Cases | Severe Cases | Death |
| --- | --- | --- | --- | --- |
| 2022/02/01 | 7 | 0 | 0 | 0 |
|  | 5 | 18.953 | 30.94 | 31.168 |
|  | 3 | 22.096 | 41.191 | 42.256 |
|  | 0 | 27.836 | 61.436 | 64.212 |
| 2022/04/17 | 7 | 0 | 0 | 0 |
|  | 5 | 11.783 | 10.103 | 9.4061 |
|  | 3 | 15.852 | 16.792 | 16.157 |
|  | 0 | 23.372 | 29.912 | 29.387 |
| 2022/06/21 | 7 | 0 | 0 | 0 |
|  | 5 | 6.8044 | 3.7333 | 3.2896 |
|  | 3 | 10.855 | 10.035 | 9.8191 |
|  | 0 | 18.246 | 22.129 | 22.336 |
| 2022/08/29 | 7 | 0 | 0 | 0 |
|  | 5 | 4.1619 | 2.3935 | 1.9772 |
|  | 3 | 8.9826 | 9.1502 | 8.4419 |
|  | 0 | 17.935 | 22.29 | 21.049 |

**Supplementary Table 6.** Cumulative number of confirmed cases, number of critically ill patients, and number of deaths since the date of isolation relaxation due to reduction in quarantine period (simulation period: 6 months from the specified date)

| Date | Quarantine duration | Confirmed Cases | Severe Cases | Death |
| --- | --- | --- | --- | --- |
| 2022/02/01 | 7 | 1.64E+07 | 10367 | 3576.9 |
|  | 5 | 1.70E+07 | 11504 | 3905.3 |
|  | 3 | 1.76E+07 | 12650 | 4302 |
|  | 0 | 1.87E+07 | 15192 | 5210.6 |
| 2022/04/17 | 7 | 6.36E+06 | 14198 | 6006.4 |
|  | 5 | 6.76E+06 | 15034 | 6321.9 |
|  | 3 | 6.97E+06 | 15806 | 6636.5 |
|  | 0 | 7.40E+06 | 17401 | 7281.5 |
| 2022/06/21 | 7 | 6.29E+06 | 16871 | 6930 |
|  | 5 | 6.62E+06 | 17343 | 7089 |
|  | 3 | 6.90E+06 | 18488 | 7564.4 |
|  | 0 | 7.43E+06 | 20696 | 8495.1 |
| 2022/08/29 | 7 | 5.60E+06 | 17654 | 7778 |
|  | 5 | 5.89E+06 | 18172 | 7967 |
|  | 3 | 6.15E+06 | 19351 | 8465.3 |
|  | 0 | 6.64E+06 | 21634 | 9434.7 |

**Supplementary Table 7.** Ratio of increase in the number of confirmed cases, the number of critically ill patients, and the number of deaths since the date of quarantine relaxation due to the reduction of the quarantine period (period: 6 months from the specified date)

| Date | Quarantine duration | Confirmed Cases | Severe Cases | Death |
| --- | --- | --- | --- | --- |
| 2022/02/01 | 7 | 0 | 0 | 0 |
|  | 5 | 3.9979 | 10.97 | 9.1801 |
|  | 3 | 7.4031 | 22.023 | 20.271 |
|  | 0 | 13.944 | 46.547 | 45.673 |
| 2022/04/17 | 7 | 0 | 0 | 0 |
|  | 5 | 6.2814 | 5.8873 | 5.2517 |
|  | 3 | 9.6573 | 11.325 | 10.489 |
|  | 0 | 16.359 | 22.564 | 21.228 |
| 2022/06/21 | 7 | 0 | 0 | 0 |
|  | 5 | 5.1155 | 2.7966 | 2.2936 |
|  | 3 | 9.6677 | 9.5834 | 9.1542 |
|  | 0 | 17.966 | 22.668 | 22.584 |
| 2022/08/29 | 7 | 0 | 0 | 0 |
|  | 5 | 5.1314 | 2.9351 | 2.4287 |
|  | 3 | 9.8331 | 9.6161 | 8.8357 |
|  | 0 | 18.493 | 22.548 | 21.299 |


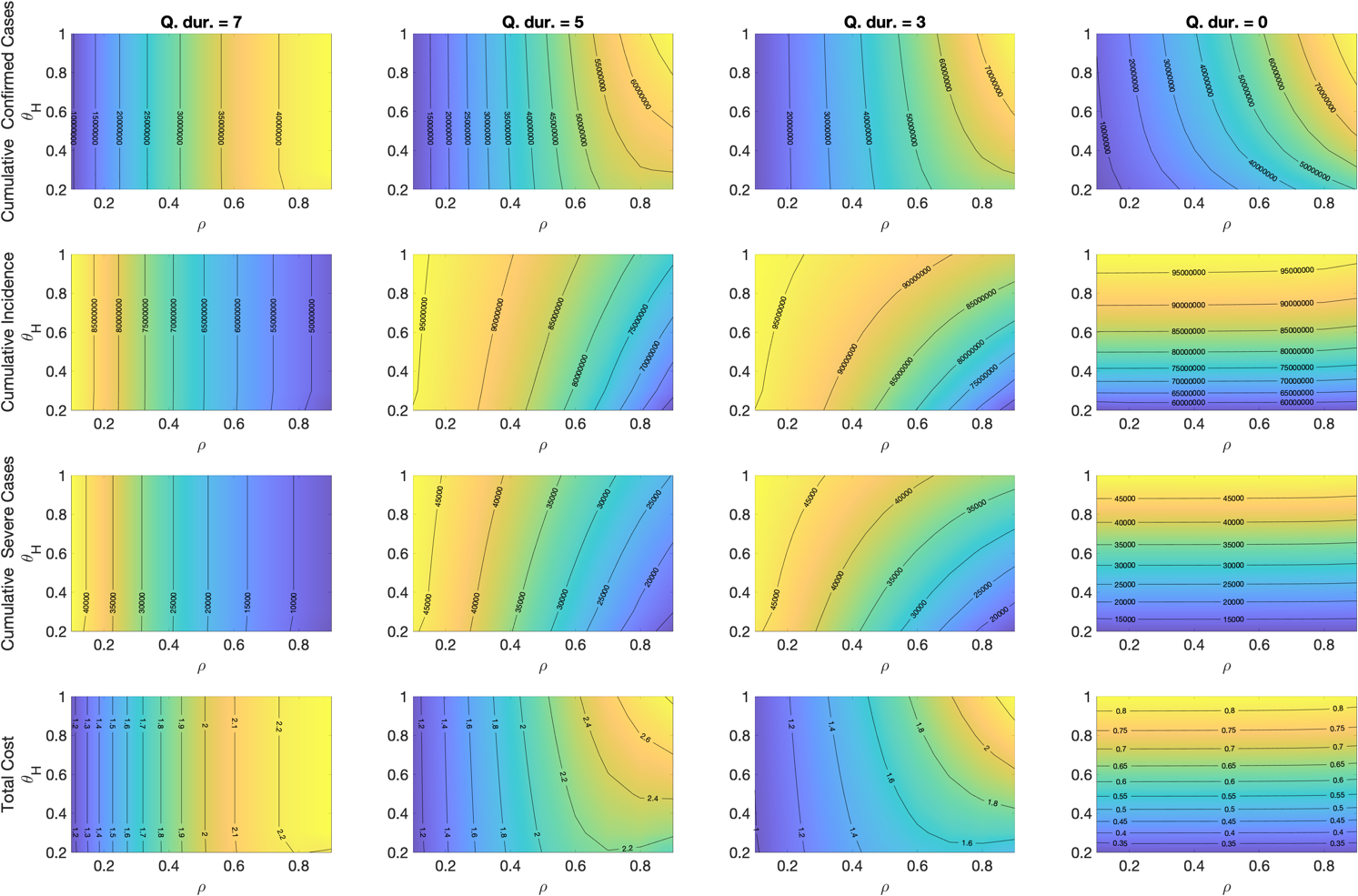


**Supplementary Figure 4.** The effects of changes in screening rates and activity in early releasers for (A) cumulative confirmed cases (B) cumulative Incidence (C) cumulative severe symptom cases and (D) Total cost $\times{10}^{-10}$ (The start dates of shortening the quarantine duration: February 1, 2022)


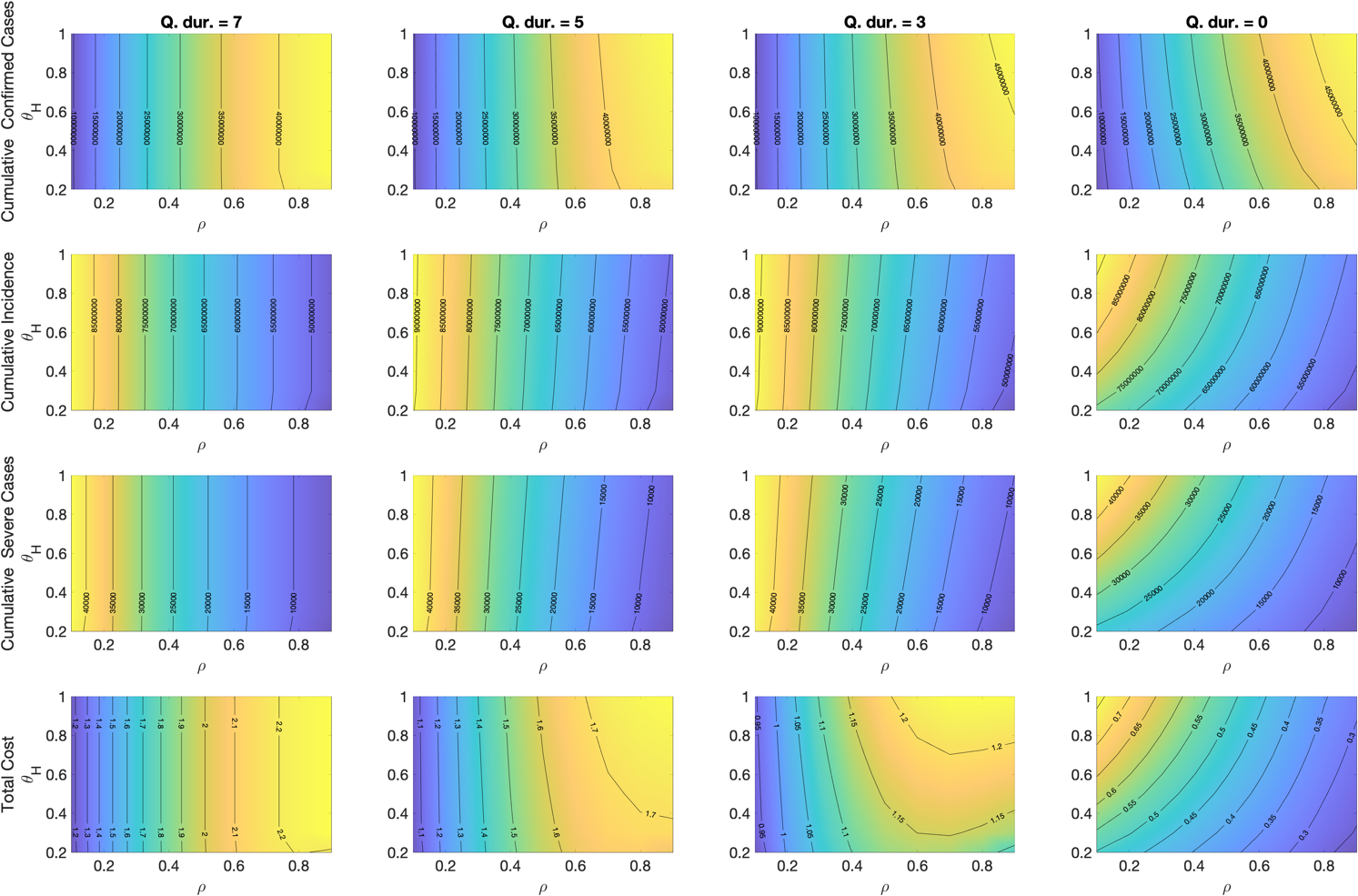


**Supplementary Figure 5**. The effects of changes in screening rates and activity in early releasers for (A) cumulative confirmed cases (B) cumulative Incidence (C) cumulative severe symptom cases and (D) Total cost $\times{10}^{-10}$ (The start dates of shortening the quarantine duration: April 17, 2022)


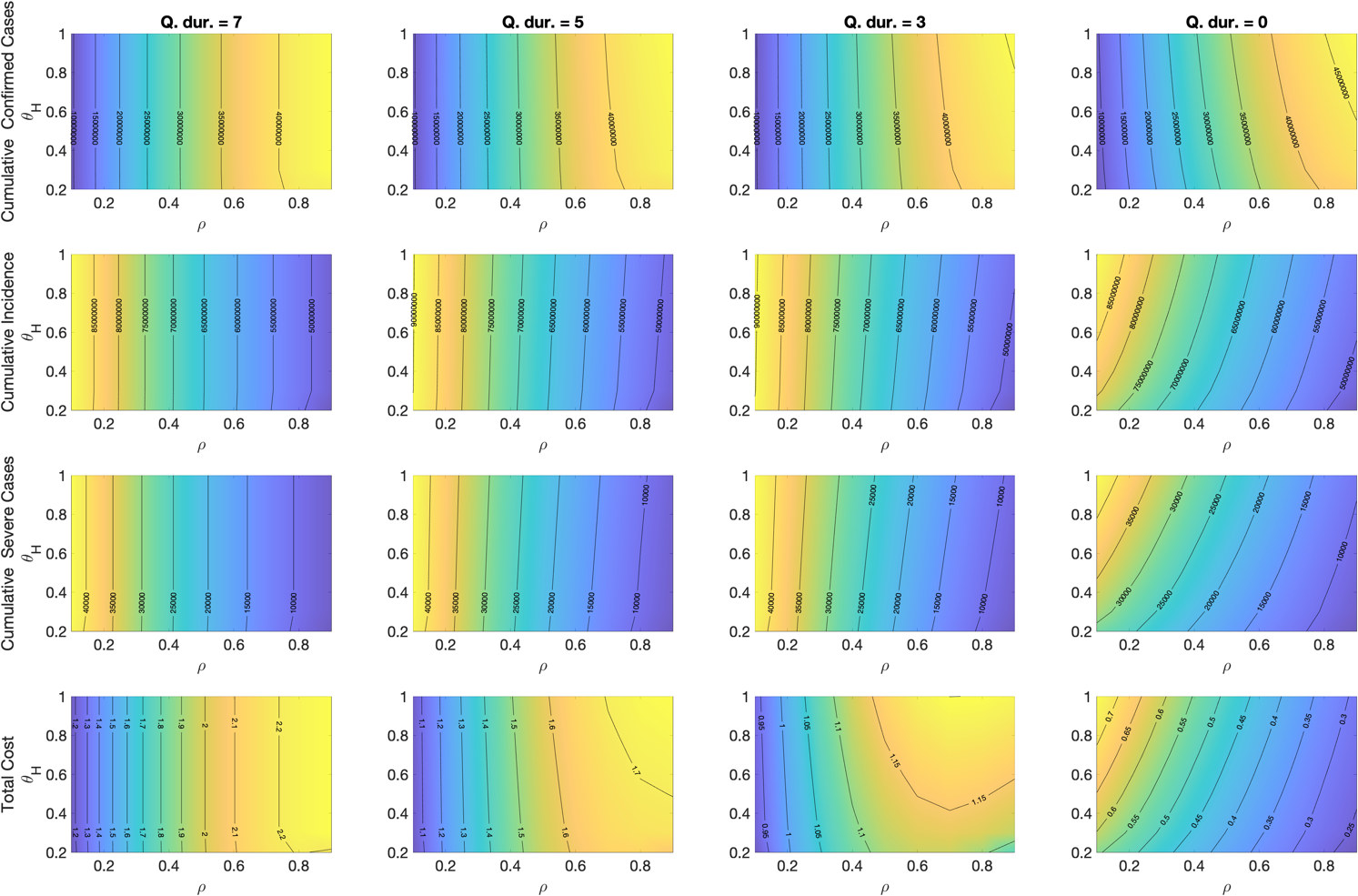


**Supplementary Figure 6.** The effects of changes in screening rates and activity in early releasers for (A) cumulative confirmed cases (B) cumulative Incidence (C) cumulative severe symptom cases and (D) Total cost $\times{10}^{-10}$ (The start dates of shortening the quarantine duration: June 21, 2022)


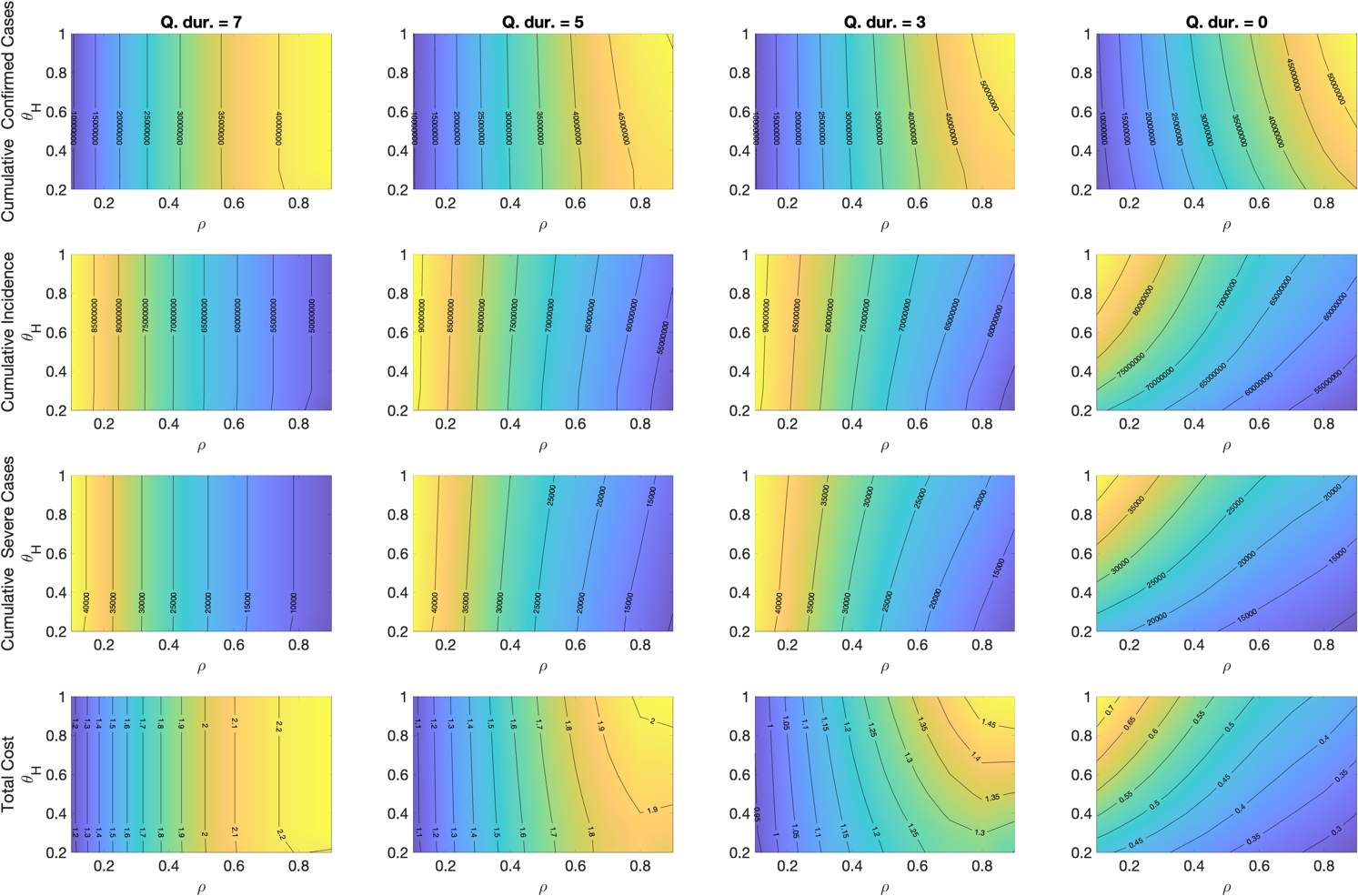


**Supplementary Figure 7.** The effects of changes in screening rates and activity in early releasers for (A) cumulative confirmed cases (B) cumulative Incidence (C) cumulative severe symptom cases and (D) Total cost $\times{10}^{-10}$ (The start dates of shortening the quarantine duration: August 29, 2022)

# Mask Policy

## Mask mandatory policy

**Supplementary Table 8.** Changes in the mandatory mask wearing policy in Korea

| Date | Policy description | Reference |
| --- | --- | --- |
| April 12, 2021 | The mandatory to wear indoor masks is started regardless of the level of social distancing.  Even outdoor, wearing masks is mandatory if a distance of 2 meters is not maintained. | (11) |
| September 26, 2022 | The mandatory to wear outdoor masks is lifted and switched to wearing masks outdoor recommendations. | (12) |
| January 30, 2023 | The mandatory to wear indoor masks is lifted and switched to wearing masks recommendations.  However, it is mandatory to wear masks in facilities that vulnerable to infection, medical facilities, pharmacies, and public transportation. | (13) |

## Lifting indoor masks policy

The Korea government decided to discuss lifting the mandatory wearing indoor masks through discussions with experts when two of the four criteria in the table below are met.

**Supplementary Table 9.** Criteria for removal of indoor mask duty in Korea

| Criteria | Evaluation items | Reference value | Reference |
| --- | --- | --- | --- |
| Stabilization of the number of new confirmed cases | Weekly new confirmed cases | Decrease for two weeks in-a-row | (14) |
| Reduction of the number of new severe cases and deaths | Weekly new severe cases | Decrease compared to previous week |  |
|  | Weekly mortality rate | Less than 0.10% |  |
| Stabilization of capability of medical response | Availability of intensive care beds available within 4 weeks | More than 50% |  |
| Obtain immunity for high-risk group | Vaccination rate of elderly group in winter | More than 50% |  |
|  | Vaccination rate of high-risk group in winter | More than 60% |  |
| Additional criteria: there is no concern that emergence of new variant or explosion of new confirmed cases according to overseas situation. | | |  |

#
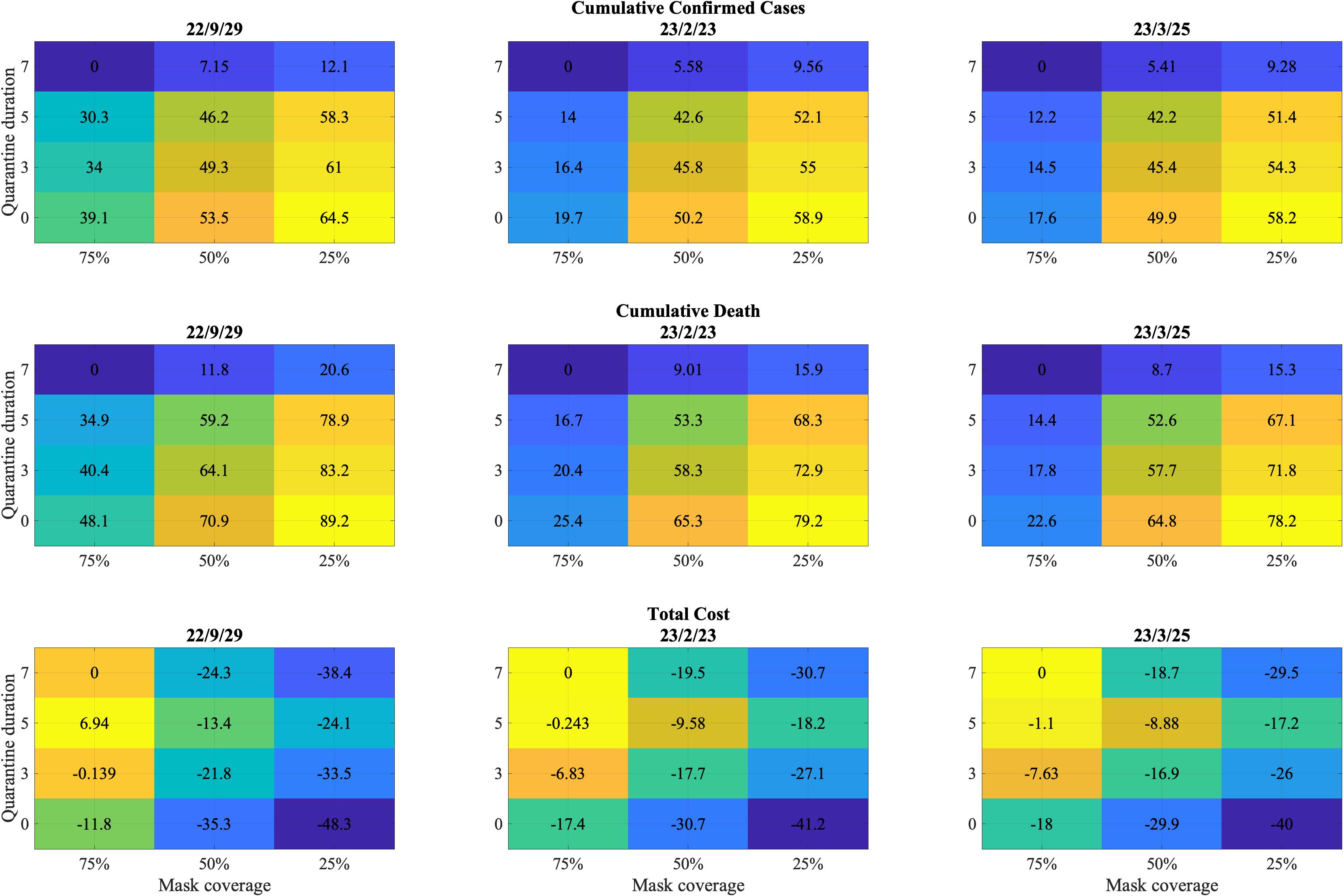
Effect of removal of indoor mask duty

**Supplementary Figure 8.** The rate of change in the cumulative number of confirmed cases, the cumulative number of severely ill patients, and total cost according to the quarantine period and mask coverage rate

**Supplementary Table 10.** Number of confirmed cases, number of critically ill patients, number of deaths, total cost of each factor

| Date | Quarantine duration | Mask Coverage | Confirmed cases | | Severe symptom cases | Death | Total cost | (Medical expenses) | (Wage loss) | (Mask cost) |
| --- | --- | --- | --- | --- | --- | --- | --- | --- | --- | --- |
| 2022/09/29 | 7 | 75% | 1.88E+07 | 12497 | | 4564.7 | 3.93E+12 | 1.73E+09 | 4.84E+09 | 3.92E+12 |
|  |  | 50% | 2.22E+07 | 15857 | | 5743.9 | 1.75E+12 | 2.15E+09 | 5.78E+09 | 1.74E+12 |
|  |  | 25% | 2.42E+07 | 18049 | | 6558.5 | 4.44E+11 | 2.41E+09 | 6.32E+09 | 4.35E+11 |
|  | 5 | 75% | 2.13E+07 | 14359 | | 5166.4 | 3.93E+12 | 1.98E+09 | 3.97E+09 | 3.92E+12 |
|  |  | 50% | 2.63E+07 | 19061 | | 6821.5 | 1.75E+12 | 2.56E+09 | 4.94E+09 | 1.74E+12 |
|  |  | 25% | 2.96E+07 | 22452 | | 8045.7 | 4.44E+11 | 2.97E+09 | 5.58E+09 | 4.35E+11 |
|  | 3 | 75% | 2.31E+07 | 15937 | | 5714 | 3.92E+12 | 2.18E+09 | 2.67E+09 | 3.92E+12 |
|  |  | 50% | 2.77E+07 | 20478 | | 7330.4 | 1.75E+12 | 2.74E+09 | 3.23E+09 | 1.74E+12 |
|  |  | 25% | 3.07E+07 | 23684 | | 8483.3 | 4.42E+11 | 3.12E+09 | 3.60E+09 | 4.35E+11 |
|  | 0 | 75% | 2.54E+07 | 18090 | | 6473.7 | 3.92E+12 | 2.45E+09 | 2.42E+08 | 3.92E+12 |
|  |  | 50% | 2.95E+07 | 22312 | | 7987.8 | 1.75E+12 | 2.95E+09 | 2.97E+08 | 1.74E+12 |
|  |  | 25% | 3.22E+07 | 25381 | | 9080.2 | 4.39E+11 | 3.32E+09 | 3.38E+08 | 4.35E+11 |
| 2023/02/23 | 7 | 75% | 1.88E+07 | 12497 | | 4564.7 | 3.93E+12 | 1.73E+09 | 4.84E+09 | 3.92E+12 |
|  |  | 50% | 2.11E+07 | 14639 | | 5327.9 | 2.63E+12 | 2.00E+09 | 5.46E+09 | 2.62E+12 |
|  |  | 25% | 2.23E+07 | 15884 | | 5767.5 | 1.86E+12 | 2.15E+09 | 5.79E+09 | 1.85E+12 |
|  | 5 | 75% | 1.91E+07 | 12729 | | 4638 | 3.92E+12 | 1.76E+09 | 3.56E+09 | 3.92E+12 |
|  |  | 50% | 2.46E+07 | 17312 | | 6210.6 | 2.63E+12 | 2.35E+09 | 4.59E+09 | 2.62E+12 |
|  |  | 25% | 2.66E+07 | 19291 | | 6899.4 | 1.86E+12 | 2.59E+09 | 4.99E+09 | 1.85E+12 |
|  | 3 | 75% | 2.01E+07 | 13623 | | 4949.1 | 3.92E+12 | 1.88E+09 | 2.32E+09 | 3.92E+12 |
|  |  | 50% | 2.61E+07 | 18761 | | 6719.3 | 2.63E+12 | 2.53E+09 | 3.03E+09 | 2.62E+12 |
|  |  | 25% | 2.80E+07 | 20688 | | 7389.6 | 1.85E+12 | 2.76E+09 | 3.26E+09 | 1.85E+12 |
|  | 0 | 75% | 2.13E+07 | 14769 | | 5348.3 | 3.92E+12 | 2.02E+09 | 1.97E+08 | 3.92E+12 |
|  |  | 50% | 2.80E+07 | 20693 | | 7402.5 | 2.63E+12 | 2.76E+09 | 2.76E+08 | 2.62E+12 |
|  |  | 25% | 2.98E+07 | 22561 | | 8049 | 1.85E+12 | 2.99E+09 | 3.00E+08 | 1.85E+12 |
| 2023/03/25 | 7 | 75% | 1.88E+07 | 12497 | | 4564.7 | 3.93E+12 | 1.73E+09 | 4.84E+09 | 3.92E+12 |
|  |  | 50% | 2.11E+07 | 14708 | | 5357 | 2.77E+12 | 2.01E+09 | 5.47E+09 | 2.76E+12 |
|  |  | 25% | 2.23E+07 | 15847 | | 5771.2 | 2.08E+12 | 2.15E+09 | 5.78E+09 | 2.07E+12 |
|  | 5 | 75% | 1.90E+07 | 12663 | | 4623.7 | 3.92E+12 | 1.76E+09 | 3.55E+09 | 3.92E+12 |
|  |  | 50% | 2.46E+07 | 17305 | | 6215.2 | 2.77E+12 | 2.35E+09 | 4.59E+09 | 2.76E+12 |
|  |  | 25% | 2.64E+07 | 19080 | | 6845.9 | 2.08E+12 | 2.57E+09 | 4.95E+09 | 2.07E+12 |
|  | 3 | 75% | 1.99E+07 | 13499 | | 4915.9 | 3.92E+12 | 1.86E+09 | 2.30E+09 | 3.92E+12 |
|  |  | 50% | 2.60E+07 | 18718 | | 6712.7 | 2.77E+12 | 2.52E+09 | 3.02E+09 | 2.76E+12 |
|  |  | 25% | 2.77E+07 | 20414 | | 7313.7 | 2.07E+12 | 2.73E+09 | 3.23E+09 | 2.07E+12 |
|  | 0 | 75% | 2.10E+07 | 14540 | | 5281.6 | 3.92E+12 | 1.99E+09 | 1.94E+08 | 3.92E+12 |
|  |  | 50% | 2.79E+07 | 20603 | | 7379.9 | 2.76E+12 | 2.75E+09 | 2.75E+08 | 2.76E+12 |
|  |  | 25% | 2.94E+07 | 22221 | | 7947.1 | 2.07E+12 | 2.95E+09 | 2.96E+08 | 2.07E+12 |

**Table S11.** The rate of change in the number of confirmed cases, the number of critically ill patients, the number of deaths, and the total cost

| Date | Quarantine duration | Mask Coverage | Confirmed cases | Severe symptom cases | Death | Total Cost |
| --- | --- | --- | --- | --- | --- | --- |
| 2022/09/29 | 7 | 75% | 0 | 0 | 0 | 0 |
|  |  | 50% | 18.497 | 26.891 | 25.834 | -55.428 |
|  |  | 25% | 29.068 | 44.432 | 43.68 | -88.685 |
|  | 5 | 75% | 13.653 | 14.903 | 13.182 | -0.015947 |
|  |  | 50% | 40.33 | 52.53 | 49.441 | -55.439 |
|  |  | 25% | 57.53 | 79.664 | 76.262 | -88.69 |
|  | 3 | 75% | 22.999 | 27.529 | 25.179 | -0.044065 |
|  |  | 50% | 47.717 | 63.867 | 60.589 | -55.478 |
|  |  | 25% | 63.458 | 89.52 | 85.848 | -88.736 |
|  | 0 | 75% | 35.127 | 44.76 | 41.823 | -0.099041 |
|  |  | 50% | 56.909 | 78.545 | 74.991 | -55.547 |
|  |  | 25% | 71.427 | 103.1 | 98.924 | -88.814 |
| 2023/02/23 | 7 | 75% | 0 | 0 | 0 | 0 |
|  |  | 50% | 12.203 | 17.146 | 16.721 | -32.951 |
|  |  | 25% | 18.798 | 27.107 | 26.351 | -52.723 |
|  | 5 | 75% | 1.6105 | 1.8595 | 1.6059 | -0.031782 |
|  |  | 50% | 30.967 | 38.536 | 36.059 | -32.964 |
|  |  | 25% | 41.856 | 54.372 | 51.147 | -52.732 |
|  | 3 | 75% | 6.8607 | 9.0147 | 8.4217 | -0.060546 |
|  |  | 50% | 38.908 | 50.128 | 47.202 | -33 |
|  |  | 25% | 49.163 | 65.549 | 61.887 | -52.772 |
|  | 0 | 75% | 13.389 | 18.186 | 17.169 | -0.11107 |
|  |  | 50% | 49.046 | 65.587 | 62.169 | -33.064 |
|  |  | 25% | 58.583 | 80.531 | 76.332 | -52.842 |
| 2023/03/25 | 7 | 75% | 0 | 0 | 0 | 0 |
|  |  | 50% | 12.562 | 17.698 | 17.358 | -29.456 |
|  |  | 25% | 18.602 | 26.81 | 26.433 | -47.131 |
|  | 5 | 75% | 1.1565 | 1.3309 | 1.2924 | -0.032382 |
|  |  | 50% | 30.919 | 38.48 | 36.158 | -29.469 |
|  |  | 25% | 40.692 | 52.677 | 49.976 | -47.142 |
|  | 3 | 75% | 6.0479 | 8.0221 | 7.6949 | -0.061396 |
|  |  | 50% | 38.661 | 49.787 | 47.059 | -29.505 |
|  |  | 25% | 47.69 | 63.356 | 60.224 | -47.182 |
|  | 0 | 75% | 11.968 | 16.349 | 15.707 | -0.11189 |
|  |  | 50% | 48.548 | 64.864 | 61.675 | -29.569 |
|  |  | 25% | 56.811 | 77.811 | 74.1 | -47.251 |

# Sensitivity Analysis

A sensitivity analysis is performed on the model parameters. The normalized forward sensitivity index of the cumulative incidence (${CI}_{p}$) is defined as follows (15):

${CI}_{p}=\frac{\partial(CI)}{\partial p}\times\frac{p}{CI}$.

We randomly choose a total of 100 sets from a uniform distribution in the range of $\pm10\%$ of some parameter values in Table 1 in the main manuscript. The cumulative incidence is computed for one year and the quarantine duration is fixed as 7 days. Supplementary Figure 9 illustrates the normalized forward sensitivity index of the CI with respect to the parameters. The results show that the transmission rate (*β*) is the most positive effect on the CI, while the screening rate for confirmation of patients ($\rho$) is the most negative effect on the $CI$. In addition, the third-dose vaccine efficacy ($\tau_{3}$) and the fourth-dose vaccine efficacy ($\tau_{4}$) are negative influential parameters, which implies that if the efficacy of booster vaccination increases, the cumulative incidence will decrease.


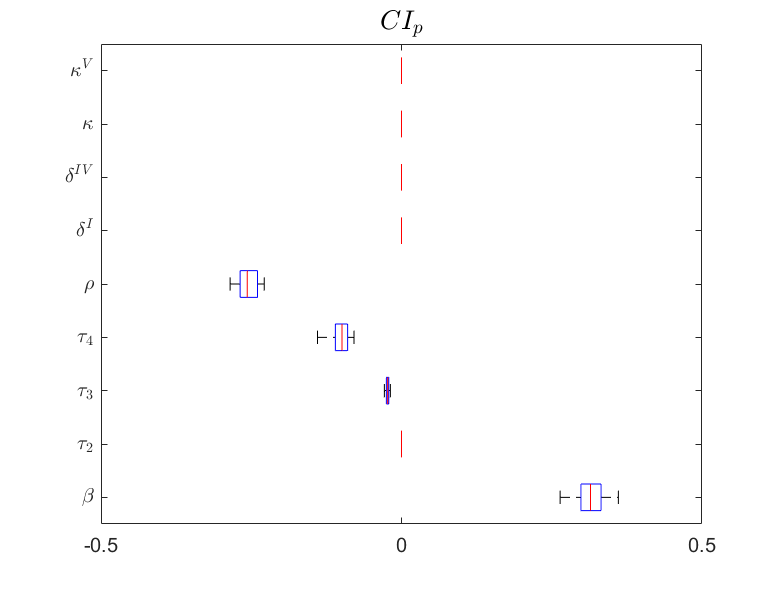


**Supplementary Figure 9.** Normalized forward sensitivity index of the CI on the parameters $\beta$, $\tau_{2}$, $\tau_{3}$, $\tau_{4}$, $\rho$, $\delta_{i}$, $\delta_{i}^{V}$, $\kappa_{i}$, and $\kappa_{i}^{V}$

# Reference

1. Korea Disease Control and Prevention Agency (KDCA). The regular briefing om July 6^th^ . <https://www.kdca.go.kr/board/board.es?mid=a20501010000&bid=0015&list_no=720037&cg_code=&act=view&nPage=65>. [Accessed on June 21, 2023].
2. Korea Disease Control and Prevention Agency (KDCA). The regular briefing om August 2^nd^. <https://www.kdca.go.kr/board/board.es?mid=a20501010000&bid=0015&list_no=720313&cg_code=&act=view&nPage=5>. [Accessed on June 21, 2023].
3. Korea Disease Control and Prevention Agency (KDCA). The regular briefing om August 30^th^. <https://www.kdca.go.kr/board/board.es?mid=a20501010000&bid=0015&list_no=720575&cg_code=&act=view&nPage=5>. [Accessed on June 21, 2023].
4. Goyal A, Reeves DB, Thakkar N, Famulare M, Cardozo-Ojeda EF, Mayer BT, et al. Slight reduction in SARS-CoV-2 exposure viral load due to masking results in a significant reduction in transmission with widespread implementation. Scientific Reports (2021) 11(1):1–12. doi: https://doi.org/10.1038/s41598-021-91338-5
5. news The Voice for healthcare. COVID-19 treatment cost 1.13trillion won… 75.1% of health insurance financial statements. <https://www.newsthevoice.com/news/articleView.html?idxno=21357> [Accessed on February 15, 2023].
6. Health Insurance Review & Assessment Service. Information on application standards and claim method for medical care benefits for residents of the COVID-19 Living Treatment Center. <https://www.hira.or.kr/bbsDummy.do?pgmid=HIRAA020002000100&brdScnBltNo=4&brdBltNo=8499#none> [Accessed on February 15, 2023].
7. Employed persons by gender/ age group. <https://kosis.kr/statHtml/statHtml.do?orgId=101&tblId=DT_1DA7024S&vw_cd=MT_ETITLE&list_id=B17&scrId=&language=en&seqNo=&lang_mode=en&obj_var_id=&itm_id=&conn_path=MT_ETITLE&path=%252Feng%252FstatisticsList%252FstatisticsListIndex.do>
8. Age, Days, Hours, Payments, Workers by Size, Education, Age. <https://kosis.kr/statHtml/statHtml.do?orgId=118&tblId=DT_PAY0004&vw_cd=MT_ETITLE&list_id=D_6&scrId=&language=en&seqNo=&lang_mode=en&obj_var_id=&itm_id=&conn_path=MT_ETITLE&path=%252Feng%252FstatisticsList%252FstatisticsListIndex.do>
9. World Bank. The cost of air pollution: strengthening the economic case for action. (2016). doi: <https://doi.org/10.1596/25013>
10. Ministry of Food and Drug Safety. Supply and demand trends such as mask production. <https://www.mfds.go.kr/brd/m_99/view.do?seq=46218&srchFr=&srchTo=&srchWord=%EB%A7%88%EC%8A%A4%ED%81%AC&srchTp=0&itm_seq_1=0&itm_seq_2=0&multi_itm_seq=0&company_cd=&company_nm=&Data_stts_gubun=C9999&page=1> [Accessed on February 15, 2023].
11. Korea Disease Control and Prevention Agency (KDCA). COVID-19 outbreak and vaccination status in Korea. <https://ncov.kdca.go.kr/tcmBoardView.do?brdId=3&brdGubun=31&dataGubun=&ncvContSeq=5119&contSeq=5119&board_id=312&gubun=BDJ> [Accessed on February 15, 2023].
12. Korea Disease Control and Prevention Agency (KDCA). Voluntary conversion to wear outdoor masks and announcement of the results of a nationwide COVID-19 antibody positive rate survey. <https://ncov.kdca.go.kr/tcmBoardView.do?brdId=&brdGubun=&dataGubun=&ncvContSeq=372996&contSeq=372996&board_id=&gubun=ALL> [Accessed on February 15, 2023].
13. Korea Disease Control and Prevention Agency (KDCA). After the Lunar New Year holidays, the first phase of adjusting the mandatory wearing of masks was implemented by reviewing whether the table was met and the overseas situation. <https://ncov.kdca.go.kr/tcmBoardView.do?brdId=3&brdGubun=31&dataGubun=&ncvContSeq=7116&contSeq=7116&board_id=312&gubun=ALL> [Accessed on February 15, 2023].
14. Ministry of Health and Welfare. Change the obligation to wear a mask indoors to recommendation when trends of patients and severe patients, medical capacity, etc. are met. <https://www.mohw.go.kr/react/al/sal0301vw.jsp?PAR_MENU_ID=04&MENU_ID=0403&page=3&CONT_SEQ=374235> [Accessed on February 15, 2023].
15. Lee, H., Kim, J. E., Lee, S., & Lee, C. H. (2018). Potential effects of climate change on dengue transmission dynamics in Korea. *PLoS One*, *13*(6), e0199205.
